# RNA circularization preserves dynamics and enables in-cell relaxation NMR

**DOI:** 10.1093/nar/gkag675

**Published:** 2026-07-14

**Authors:** Henry T P Annecke, Katja Bekcic, Sabrina Toews, Hannes Feyrer, Katja Petzold

**Affiliations:** Department of Medical Biochemistry and Microbiology, Center of Excellence for the Chemical Mechanisms & Science for Life Laboratory, Uppsala University, Uppsala, 75237, Sweden; Department of Medical Biochemistry and Biophysics, Karolinska Institute, Stockholm 17177, Sweden; Department of Medical Biochemistry and Microbiology, Center of Excellence for the Chemical Mechanisms & Science for Life Laboratory, Uppsala University, Uppsala, 75237, Sweden; Department of Medical Biochemistry and Biophysics, Karolinska Institute, Stockholm 17177, Sweden; Department of Medical Biochemistry and Biophysics, Karolinska Institute, Stockholm 17177, Sweden; Department of Medical Biochemistry and Microbiology, Center of Excellence for the Chemical Mechanisms & Science for Life Laboratory, Uppsala University, Uppsala, 75237, Sweden; Department of Medical Biochemistry and Biophysics, Karolinska Institute, Stockholm 17177, Sweden

## Abstract

RNA function is governed by transient structural rearrangements that are sensitive to the cellular environment. While NMR spectroscopy provides unique access to RNA dynamics at atomic resolution, such measurements are typically restricted to *in vitro* conditions due to rapid degradation of unmodified RNA by intracellular RNases. Here, we develop a modular and efficient enzymatic pipeline for circularizing hairpins to enhance stability and enable in-cell NMR studies of RNA dynamics. The circular RNA exhibits a lifetime of >24 h in lysate, comparable to fully 2′-*O*-methylated RNA, without requiring chemical modifications. Circularization also preserves the secondary structure of four biologically and structurally diverse RNAs as well as maintaining native excited-state dynamics for two of the constructs. Interestingly, circularization of RNAs whose dynamics involve interhelical bending reveals a transfer of dynamics across RNA helices, reminiscent of allosteric cooperativity. The enhanced intracellular stability enables the first reproducible in-cell measurements of imino proton relaxation rates on chemically unmodified RNA. Together, this work establishes RNA circularization as a broadly applicable strategy for quantitative in-cell NMR studies of RNA structure and dynamics, and uncovers a possible mechanism for long-range cooperativity in RNA.

## Graphical Abstract

**Graphical Abstract:**
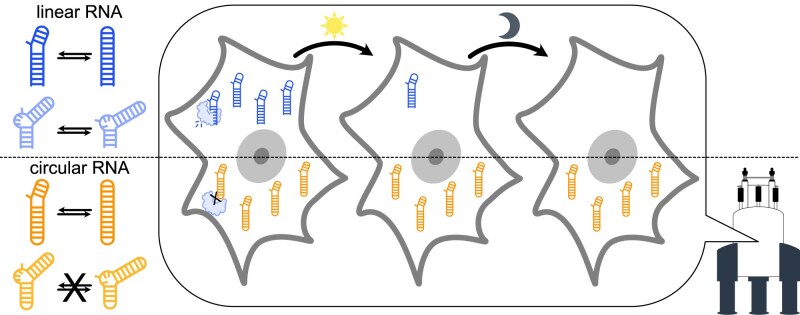

## Introduction

RNA is a versatile biomolecule that performs vital functions, including regulating gene expression at both transcriptional and post-transcriptional levels [1–4]. Like other functional molecules, RNA undergoes structural changes, known as dynamics, on a range of timescales to perform its functions. The lowest energy conformation, known as the ground state (GS), is in exchange with one or more higher energy excited state (ES) conformations, and dynamics occurring on the micro- to millisecond timescale have been shown to have significant cellular impact [5–12]. The most suitable method to reveal RNA dynamics on this timescale at atomic resolution is nuclear magnetic resonance (NMR) relaxation [13–16].

While NMR can provide useful insights regarding RNA, these NMR experiments are typically carried out within simplified buffered solutions that differ from the native environment of the cell. Cells contain, for example, large macromolecular complexes as well as pH and ion gradients, which can alter RNA dynamics and are difficult to recapitulate [17–23]. There is, therefore, a growing interest in in-cell NMR to more accurately capture the biologically relevant structural ensemble of RNA [11–13, 15–17]. While previous in-cell NMR studies of RNA structure have been reported, quantitative relaxation measurements on chemically unmodified RNA have not been feasible due to rapid intracellular degradation. The study of dynamics by in-cell NMR is additionally complicated by shortened cellular viability and low signal sensitivity [23–29]. One technique to improve the viability of cells during in-cell NMR experiments is the use of bioreactors to maintain cell metabolic activity for up to 72 h [22, 30–32]. However, even with the use of a bioreactor the experimental time is limited by degradation of the intracellular RNA. To allow for faster acquisition within this limited timeframe and improve the sensitivity of in-cell NMR of nucleic acids, SOFAST techniques have been used to exploit the reduced intracellular imino T_1_ of DNA [33]. Alternatively, dynamic nuclear polarization-assisted (DNP) magic angle spinning (MAS) solid-state NMR has also been used to overcome the low sensitivity of solution state NMR of nucleic acids [26, 34]. However, solid-state DNP cannot be used to determine exchange rates associated with RNA dynamics, which necessitates a combination of multiple approaches and means addressing the limitation of RNA sample degradation. Despite advances in in-cell NMR of RNA, quantitative relaxation measurements on chemically unmodified RNA remain inaccessible due to rapid degradation.

The lifetime of RNA can be extended by chemical modifications such as 2′-*O*-methylation (methoxy) and phosphorothioate backbones, both of which have been used for in-cell NMR [22, 25, 28]. However, these modifications have been shown to alter the structure and interactions of nucleic acids, limiting their utility in the pursuit of studying intracellular RNA dynamics [35–38]. Alternatively, circular RNA (circRNA), which is also referred to as dumbbell or cyclic RNA, does not require modified RNA nucleotides and has been shown to significantly increase the intracellular half-life [39, 40]. NMR studies of RNAs often use hairpin motifs for construct design, for example in studies of the bacterial ribosomal A-site, HIV TAR, HBV, Xist tetraloop, and SARS-CoV-2 SL5 [10, 41–44]). Previous studies of circRNA found minimal impact on RNA structure and protein recognition, indicating their potential for studying RNA structure and dynamics with in-cell NMR [45–48].

In this work, we determine relaxation rates in cells for the first time, on chemically unmodified RNA, paving the way for future dynamics studies. We establish a modular enzymatic method to produce high purity circRNA for use in various applications, including in-cell NMR. The production of circRNA is achieved using either tandem repeat or conventional *in vitro* transcription (IVT) methods, to synthesize constructs with stable tetraloops, capable of efficient self-splinted ligation by T4 RNA ligase 2 [40, 49]. This method of circularization is applied to four different well-characterized, biologically diverse and dynamic RNA hairpin sequences, and its modular nature means it can readily be applied to other hairpins [10, 41, 50]. We show the circRNA produced maintains the secondary structure of their linear counterparts, preserves the dynamics of two of the RNAs, whilst revealing a mechanism for dynamic cooperativity in the remaining two. Furthermore, we show that closing the RNA hairpin with an additional tetraloop increases the half-life from 1 h to over 24 h in cell lysate. Enhancing the RNA stability allows us to determine quantitative relaxation rates inside the cell of hydrogen bonded imino protons using unmodified RNA, which, under biological replica, are revealed to be increased in the cellular environment.

## Materials and methods

Extended Materials and methods are found in Supplementary Information section Supporting Materials and methods.

### Tandem repeat *in vitro* transcription

Tandem copies of template sequences for circH44-top (20×) were inserted into pUC19 according to Feyrer *et al*. [49], and then linearized using BamHI (Thermofisher, ER0002) at 200 ng/µl. Next, RNase H (Protein Science Facility, Scilifelab) was added to the tandem IVT reaction alongside a 14 nt chimeric guide mCmGmAmA^GGTTmAmAmGmCmGmC, in which ^ represents the cleavage site on the complementary RNA strand. The reaction contained 100 mM Tris–glutamate pH 8, 9 mM Mg(OAc)_2_, 10 mM Dithiothreitol (DTT), 25 mM spermidine, 5 mM GMP, 3 mM rNTPs, 5 ng/µl linear plasmid, 20 µM chimera guide, 0.8 mg/ml T7 polymerase WT (Protein Science Facility, Scilifelab), and 0.005 mg/ml RNase H (Protein Science Facility, Scilifelab). One hundred millilitres of reaction was incubated in 12.5 ml aliquots at 37°C in a waterbath for 16 h and quenched with 20% v/v 500 mM EDTA pH 8. Reaction was concentrated in Amicon centrifugal filter with 3 kDa cutoff before gel purification.

### Ligation

Buffer exchanged RNAs were folded at 12 µM in H_2_O in 1 ml aliquots by heating to 95°C for 5 min, followed by incubation on ice for 20 min. 10 µM RNA was ligated with 1 µM T4 RNA Ligase 2 (P32277, Protein Science Facility, SciLifelab) in 50 mM Tris–HCl pH 7.5, 2 mM MgCl_2_, 1 mM DTT, and 400 μM ATP at room temperature for 3 h. Ligation was confirmed by dPAGE and quenched by freezing to −20°C.

### Solution state NMR sample preparation

Gel purified RNAs were buffer exchanged to NMR buffer (15 mM Na_3_PO_4_, 25 mM NaCl, and 0.1 mM EDTA) at pH 6.5 for MiR34a:Sirt1, circMiR34a:Sirt1, H44-top, circH44-top, GUG, circGUG, and pH 6.9 for GU, and circGU. RNAs were folded at 100 µM for circ/MiR34a:Sirt1 and circ/GU, and 15 µM for circ/H44-top) by heating in 1 ml aliquots to 95°C for 5 min, followed by 20 min on ice. Final samples of 250 µl contained 10% D_2_O and were transferred into Shigemi tubes. Final concentrations were 600 µM for circMiR34a:Sirt1, 300 μM for circGUG, 500 µM for GU, and circGU, 750 µM for both H44-top and circH44-top.

### In-cell sample preparation

RNA purified by HPLC was folded at 50 µM in MilliQ ddH_2_O by heating in 1 ml aliquots to 95°C followed by 20 min on ice. The RNA was concentrated in 3 kDa Amicon concentrators (Millipore) to 2.9 mM.

In-cell samples were prepared as previously described [33, 51]. 8x T-175 flasks (Sarstedt 83.3912.002) of HeLa (CCL-2, ATCC) cells were cultured in DMEM High Glucose, High Pyruvate (Gibco 41966052) supplemented with 10% heat inactivated FBS (Gibco A5256701) to ∼80% confluency at 37°C, 5% CO_2_. Cells were washed with 20 ml Dulbecco's Phosphate-Buffered Saline (DPBS, Gibco 14190250) and detached with 0.05% Trypsin-EDTA (Gibco 2530005). A total of 98 × 10^6^ cells were harvested by centrifugation at 300 × *g* for 3 min and washed with 12 ml of DPBS. Viability was first assessed with Trypan Blue staining and was >90% preceding experiments.

For the first biological replicate, the cells were separated into two 50 ml falcon tubes with 16.3 million resuspended cells and another with 81.6 million, both of which were then subsequently pelleted at 300 × *g* for 3 min, and the DPBS removed. The first cell aliquot was resuspended in 400 µl of 1× electroporation buffer (140 mM NaP pH 7.0, 10 mM MgCl_2_, 5 mM KCl) containing 400 µM of RNA and 10.25 µM of fluorescein labelled RNA for flow cytometry and confocal microscopy. The second cell aliquot was resuspended in 2 ml of 1× electroporation buffer containing 400 µM RNA. Four hundred microlitres of the cell suspensions were added to 6 × 4 mm cuvettes (Cell Projects EP-104) and chilled on ice for 5 minutes. Electroporation was performed with a BTX 830 ECM electroporator (Harvard Bioscience) with a total of two square wave pulses, 100 µsec 1000 V high power pulse, followed by a 7 s delay, then another 30 ms pulse with 350 V. Cuvettes were then incubated at room temperature for 2 min, and the cell suspension was flushed out with 500 µl 37°C pre-warmed L15- medium (Gibco Leibovitz’s medium, no phenol red 21083027). The cuvette containing the fluorescently labelled RNA was collected in 7 ml (first wash). The unlabelled fraction was collected in 40 ml pre-warmed L15- (first wash). Cell debris was removed from the top of the liquid. Both cell samples were then dispersed by pipetting with 10 ml serological pipettes, and pelleted (300 × *g*, 3 min), and the supernatants (first wash) flash frozen in LN2 for RNA recovery. Cell pellets were then again resuspended in 7 and 50 ml prewarmed L15- for labelled and unlabelled samples, respectively, (second wash). A total of 500 000 cells were collected from the second wash for assessment of viability and transfection efficiency via flow cytometry (both labelled and unlabelled) and confocal microscopy (second wash fluorescent). Remaining labelled and unlabelled cells were combined and pelleted (300 × *g*, 3 min) before resuspension in 1.5 ml L15 (Sigma–Aldrich L5520) + 10% D_2_O with a 5 ml serological pipette. Final cell suspension was transferred to a 5 mm Shigemi tube (Shigemi Inc. BMS-005B) without plunger and pelleted by hand-crank centrifugation to form a fluffy pellet of approximately 400 µl.

Biological replicate 2 was then performed as above, using recovered RNA (see below), with a concentration of 320 µM recovered RNA in 1× electroporation buffer, and without subsequent separation of fluorescently labelled and unlabelled samples.

### Recovery of remaining RNA following electroporation

To recover the remaining, untransfected RNA from the first wash for use in the second biological replicate, the flash frozen first wash of 50 ml labelled and unlabelled supernatant was lyophilized and resuspended in 5 ml MilliQ H_2_O. The RNA was EtOH precipitated by addition of 500 µl of 3 M NaOAc (pH 5.5) and 15 ml of 4°C EtOH, and incubating in liquid nitrogen for 30 min. This was pelleted at 4500 × *g* for 30 min, and the final pellet was resuspended in 5 ml MilliQ H_2_O. The absence of RNase activity was tested by incubating overnight at room temperature. The final product was filtered with 0.2 µm syringe filters and HPLC purified with the same gradients as above. Final purity was assessed by denaturing polyacrylamide gel electrophoresis (dPAGE).

### Degradation assay

Cell lysate for RNA lifetime analysis was prepared from HEK293T (ATCC) cells. Cells were cultured in DMEM High Glucose, High Pyruvate (Gibco 41966052) supplemented with 10% heat inactivated FBS (Gibco A5256701) to ∼80% confluency at 37°C, 5% CO_2_. 66.4 million cells were harvested and pelleted at 300 × *g* for 3 min. The supernatant was removed and the cell pellet was subjected to cell lysis by four freeze–thaw cycles (LN2 and 37°C water bath), followed by sonication 3 × 20 s on medium setting. The lysed cells were then resuspended in 1200 µl of DPBS and pelleted at 10 000 × *g* for 5 min at 4°C to remove cell debris. Nine hundred microlitres of supernatant was split into three samples of 300 µl each, to which 1250 pmol of H44-top, methoxy H44-top, and circH44-top were added and mixed by pipetting. Samples were taken immediately (0 h) and at 1, 4, and 24 h. They were diluted 1:10 in denaturing dye mix, and flash frozen in LN2 at each time point, and stored at −80°C until dPAGE analysis. The same process was repeated for circ/GUG, circ/MiR34a:Sirt1, and circ/GU.

Quantification of RNA degradation was performed by analysing SYBR Gold stained bands in ImageLab. Error bars were determined from calibration replicates of pipetting precision from 1 to 10 μl pipette used to load gel samples. Exponential decay curves were fit with logarithmic A*exp(−*t*/Tau) + *C*, in which *C* was constrained to be < 5%. Lower AIC values were used to determine if exponential or no-decay (*y* = *a*, where *a* is a constant) fits should be used.

### NMR

For NMR experiments, a Bruker Avance III HD spectrometer with 600.85 MHz ^1^H base frequency and a 5 mm QCI cryoprobe (^1^H/^13^C/^15^N/^31^P) with *z*-gradient was used. Acquisition and analysis was performed with Bruker Topspin 3.6 software. 90^o^ pulse length was calibrated for each sample. I*n vitro* samples were shimmed using Topshim 3D. All experiments were performed at 25°C, unless specified otherwise.

### *In vitro*  ^1^H and ^15^N R_1ρ_

Isolated reporter peaks in the dynamic regions were chosen for each of the constructs. Imino ^1^H R_1ρ_ data were acquired as described [52, 53]. Eight data points per spin-lock (SL) strength for the on-resonance experiments were collected with the spin-lock (SL) centred on the peak of interest (Supplementary Table S1). Off-resonance experiments were carried out with three different SL strengths, with offsets ranging from −4 × SL to +4 × SL (Supplementary Table S1). Data were processed with 0th order baseline correction. ^1^H and ^15^N R_1ρ_ values were fit to monoexponential decay curves with noise calculated from Topspin signal-to-noise function, for 500 iterations of Monte-Carlo to estimate the R_1ρ_ error. On- and off-resonance datasets were fitted to either two-state reduced [54] or two-state models [55], with error received from Monte-Carlo 500 iterations. AICc, BIC, and F-test statistics were used to distinguish between two-state and three-state exchange models.

### Chemical shift perturbation analysis

Imino proton chemical shifts between linear and circular constructs were compared in ppm. The absolute value of their difference was determined and plotted. In order to determine the baseline chemical shift differences from small differences in sample conditions, i.e. pH, salt, temperature, the 95% confidence interval from the standard deviation in the apical UUCG-H1 between linear and circular constructs was selected.

### Selective inversion recovery

Experiments were performed as previously described [33]. Briefly, a ^1^H imino selective inversion pulse, followed by a variable delay and then a selective excitation pulse with a short filter gradient in between. All parameters are as before with following changes: Eburp and Iburp [56] carrier frequencies at 14.983 ppm, and 512 scans. *In vitro* selective inversion recovery experiments were performed with 32 scans. In-cell delays were in order: 0.32, 50, 900, 15, 0.32, 250, and 0.32 ms for biological replicate 1, and 0.32, 50, 900, 0.32, 15, and 250 ms for biological replicate 2. *In vitro* delays were 0.32, 125, 375, 500, 625, 750, 875, and 1000 ms. In-cell data were analysed with third-order baseline correction between 10.2 and 15 ppm, and 15 Hz line broadening was applied. As previously described, to reduce the impact of noise and bias in low signal-to-noise inversion recovery fitting, the chemical shift of the peaks for the shortest delay (and therefore maximum signal-to-noise, 0.32 ms) were used to measure the intensity changes of subsequent delays. For *in vitro* data third-order baseline correction was applied with 0.3 Hz line broadening. Data were fit to exponential recovery equation *I*(*t*) = *I*(0) (1 – a*exp(–*t*/selT_1_)) [33] in which *t* represents variable delay, selT_1_ the inversion recovery time, *I*(0) the intensity expected at time *t *>> selT_1_, and “a” the inversion efficiency. Global fits were performed with shared selT_1_ only. Optimal recovery times were determined as previously [33, 57] and are listed in the respective figures.

### Quantification of in-cell signal decay, supernatant signal contribution, and signal enhancement

For all quantifications, the visible in-cell imino peaks were integrated between 14 and 10 ppm. For supernatant comparisons, the spectra immediately preceding the supernatant measurements were integrated as comparison to the supernatant control signals. For in-cell signal decay, all spectra were integrated and fit to linear decay model, globally sharing the slope but not intercept. For signal enhancement quantification, the integrated imino proton regions between spectra acquired for 15 min and 21 s, and 15 min and 15 s for 100 ms recovery delay and 10 ms recovery delay spectra, respectively.

### Statistical methods

Fit errors for selT_1_ (*n *= 1 *in vitro* and *n* = 2 in-cell) and in-cell signal decay (*n* = 2) represent one standard deviation, for R_1ρ_ error was estimated from 500 Monte-Carlo iterations, see corresponding Materials and methods section above. Volume uncertainty from pipetting was calculated from replicates (*n* = 3) used in gel quantification. A total of 10 000 events were used for flow cytometry assessment of viability and transfection efficiency. Microscopy analysis for localization was performed on *n* = 14 cells. For chemical shift perturbation (CSP), the 95% confidence interval from the standard deviation in the apical UUC**G-H1** between linear and circular constructs was chosen. Replicate numbers and analyses are additionally indicated in their corresponding Materials and methods, and figure captions.

## Results

### Modular pipeline for production of circular RNA

**Figure 1.**
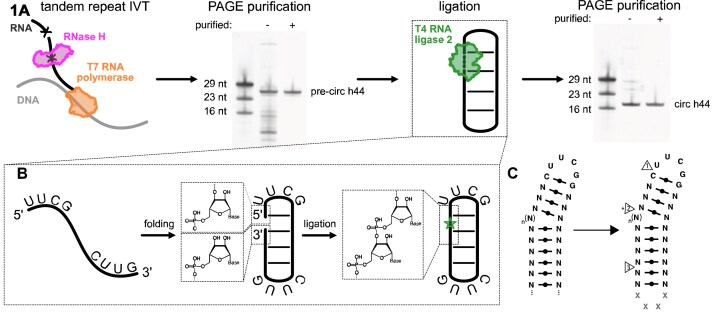
Enzymatic pipeline for high yield and purity production of circular H44-top RNA. (**A**) Tandem repeat IVT [49] does not depend on starting nucleotides and produces prominent and isolated target products with homogeneous 5′-p and 3′-OH ends. This facilitates denaturing gel purification and the subsequent ligation, as observed by dPAGE with high yields and purity of circular H44-top. (**B**) Ligation of H44-top is achieved by designing (see Box 1) the optimal ligation site proximal to the hairpin loop [40]. (**C**) Schematic scaffold design for circularization of dynamic RNA hairpins, with bulge size indicated by *n*, where *n* = 0 corresponds to no bulge. Sites for optimal ligation, in descending order, from 1, 2, and 3. Site 2 should be used in the absence of a bulge. This pipeline yields high-purity circRNA hairpins from diverse starting sequences and provides a general design framework for adapting it to other dynamic RNA motifs.

**Figure 2.**
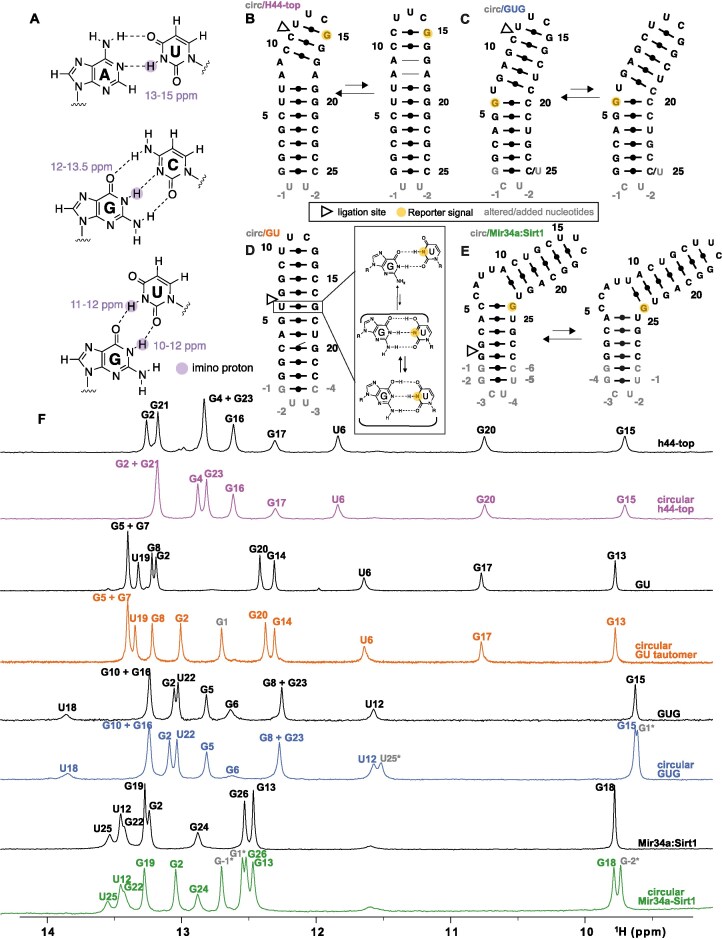
Circular constructs preserve the secondary structure of their linear counterparts, as determined by ^1^H 1D NMR. (**A**) Imino-proton base pairs observed in ^1^H 1D NMR spectra between 9 and 15 ppm, which indicate stable base pair formation. (**B–E**) Secondary structures and excited states of the circular and linear constructs (B) circ/H44-top, (C) circ/GUG, (D) circ/GU tautomer, and (E) circ/MiR34a:Sirt1 [6, 10, 52, 58]. (**F**) Imino region of ^1^H–1D spectra acquired for comparison of secondary structure. All spectra acquired at 25°C, apart from circ/GUG (black/blue) acquired at 9°C. As seen in all four biologically diverse constructs, the circularization preserves the native secondary structure, validating the strategy as suitable for downstream studies.

To protect RNA from degradation by cellular exonucleases, we developed a pipeline to enzymatically produce and circularize dynamic RNA hairpins (Figs 1A and B, and 2B–E). To test this pipeline, we chose four well characterized dynamic hairpin constructs for circularization: (i) H44-top, the tip of helix 44 in the *Escherichia coli* 30S ribosomal subunit that contains a dynamic bridge connecting the ribosomal subunits and which undergoes an apical loop rearrangement [58]; (ii) GU, representing the ubiquitous G:U wobble pair tautomeric dynamics [10]; (iii) GUG, a model system of unpaired uridine base flipping [52]; (iv) and MiR34a:Sirt1 [6], representing a miRNA:mRNA interaction in which a base pair switch from G:C to G:U extends the seed region resulting in enhanced mRNA repression (Fig. 2B–E).

In order for circularization to be suitable for structural and dynamic studies inside the cell, the existing RNA structure and dynamics must be minimally perturbed without significantly increasing the molecular size. Stable tetraloops, such as UUCG and CUUG, were chosen to close the linear hairpins, as less stable loops have been shown to destabilize the connected helix [59–63]. To increase circularization efficiency, the optimal ligation site was designed to contain at least 2 base pairs on the 3′-hydroxyl end of the precursor RNA and 0-1 base pairs on the 5′-phosphate end, as established by Chen *et al*. [40]. Therefore, for some constructs, the 5′-end of the sequences could not start with GG, which is necessary for efficient production by conventional T7 IVT (Supplementary Table S2) [64]. To circumvent this, circular H44-top (circH44-top) was synthesized by tandem IVT [49]. Additionally, tandem IVT produces the prerequisite 5′-p and 3′-OH for ligation, as well as high purity RNA (Fig. 1A and B). The terminal 5′ GG initially used in the linear construct remained in the circular constructs to facilitate structural comparison to the original constructs (Fig. 2B–E). For circular GU (circGU) and circular MiR34a:Sirt1 (circMiR34a:Sirt1), the ligation sites were altered from those recommended in Chen *et al*. [40], to enable the use of conventional IVT (Fig. 2B–E and Supplementary Fig. S1). Successful ligation of all constructs tested demonstrated the broad applicability of the method. IVT reactions were purified to remove abortive products and pre-circRNA was folded at 12 µM and ligated at 10 µM, to minimize formation of dimers during ligation, resulting in ligation efficiencies of 80% for circH44-top, 97% for circGUG, 81% for circGU, and 65% for circMiR34a:Sirt1 (Fig. 1A and B, and Supplementary Fig. S1). The ligation efficiencies remained high in constructs with altered ligation sites [40], except for MiR34a:Sirt1, where the 3′-end of the ligation site has only one base pair before the loop (Supplementary Fig. S1). The ligation was monitored by the faster migrating ligation products on dPAGE, consistent with the formation of circRNA. Later, the structure was confirmed by NMR (Figs 1A and 2F, and Supplementary Fig. S1) [40, 65]. The faster migration of circRNA also allowed for effective purification of circRNA from remaining impurities by dPAGE, resulting in isolation of pure circRNA (circH44-top 97%, circMiR34a:Sirt1 94%, circGUG 95%, and circGU 90%) (Fig 1A and Supplementary Fig. S2).

Box 1: Design summary box.

1. RNA sequence:
  - Consider how your chosen RNA motif can be inserted into the circularization template in Fig. 1C intended to keep molecular weight to a minimum to prevent unwanted NMR signal broadening or other structural alterations.
  - Ensure that your added tetraloop sits >3 bp away from the site of interest to study structure or dynamics.
  - The use of CUUG tetraloop for closing the linear RNA hairpin is recommended due to the absence of extra imino peaks at room temperature, although the UUCG tetraloop is a well-studied alternative.
2. Ligation and production:
  - Sites optimal for ligation contain at least 2 bp on the 3′-OH end of the pre-circRNA, and 0-1 on the 5′-end, this may be achieved by opening the existing apical loop; however, efficient circularization can also be achieved at other positions, Fig. 1C [40].
  - Identify whether the optimal T7 polymerase initiation sequence 5′-GG is present in your hairpin at a site suitable for ligation. If not consider the use of SPOS, tandem IVT, or 5′-ribozyme IVT [49, 66, 67].
  - Most linear RNA hairpins can be circularized with reasonable efficiency by adding two base pairs below the original 5′- and 3′-end, see ligation site 3 in Fig. 1C.
3. Controls:
  - Decide whether structure or dynamics need to be evaluated following circularization and decide on reporter peaks of interest.

### Circularization increases stability

To test whether the circularization sufficiently prolonged the lifetime of the RNA, linear, circular, and methoxy modified H44-top were incubated in HEK cell lysate over 24 h (Supplementary Fig. S3). 2′-*O*-Methylated modified RNA is commonly used in RNA drug development and in-cell NMR to improve intracellular stability based on its enhanced resistance to nuclease degradation and reduced immune response [25]. Nucleic acids were added to the lysate at an effective intracellular concentration of 37 µM, similar to quantities observed in in-cell NMR [25, 26, 68]. This concentration is sufficient for direct visualization using SYBR Gold staining for dPAGE, abolishing the need for fluorescent tags (Supplementary Fig. S3) [69].

The linear H44-top decayed to 50% within 1 h, and only ∼8% remained visible after 4 h. However, over 24 h, no significant decay is observed for either the circular or the methoxy H44-top RNA (Supplementary Fig. S3). Similarly, circGU and circ-MiR34a:Sirt1 were stabilized in HEK293T lysate with no decay observed over 24 h, whereas the linear constructs had half-lives from 30 to 60 min (Supplementary Fig. S3). Some decay is apparent in circGUG where 16% is linearized by an endonuclease after 4 h, but still demonstrates a longer half-life than linear RNA (Supplementary Fig. S3). CircH44-top was electroporated into HeLa cells [33, 51] in two biological replicas (Supplementary Figs S4–S10). In-cell NMR time-series of ^1^H-SOFAST were recorded on circH44-top and their integrals (10–14 ppm) were globally fitted between replica, indicating minimal signal reduction (−3.7 ± 1.7%/h) of the circRNA under in-cell conditions, likely due to cellular interactions (Supplementary Fig. S11). Due to the stability of circH44-top, it was possible to recover the remaining RNA after electroporation of the first in-cell NMR sample replicate by lyophilization. Followed by EtOH precipitation and subsequent HPLC purification, 639 nmol were recovered of the starting 1100 nmol (∼60% recovery yield), and recycled for the next replicate, reducing material cost.

### Circularization maintains secondary structure

To ensure that circRNAs preserve the secondary structure of their linear counterpart, ^1^H 1D imino spectra were recorded. ^1^H imino signals in the 10–15 ppm region (Fig. 2A and F) indicate the presence and structural environment of base-pairs. For all constructs tested, all base pairs found in the linear RNA were found in the circular counterpart, confirmed by ^1^H 1D imino spectra (Fig. 2F) and as assigned by ^1^H–^1^H 2D imino NOESY spectra, indicating that the circularization of all four constructs had no impact on their secondary structure (Supplementary Fig. S12), and thus can be utilized for structural studies inside the cell. Additional signals in the circular constructs result from the U:G if the UUCG tetraloop was added, and the closed terminal base pair. Furthermore, the CSP of the ^1^H imino signals between linear and circRNAs show that the base-pairs share identical chemical environments with alterations, as expected, at sites closest to the added tetraloops (Fig. 2C and Supplementary Fig. S13). For more than three base pairs away from the added nucleotides the CSP are negligible (<0.025 ppm for all constructs, Supplementary Fig. S13). This contrasts with the fully 2′-*O*-methylated modified H44-top RNA, which experienced reduced base pairing in the entire upper stem (Supplementary Fig. S14), and for which large CSP are visible for all base pairs, indicating large structural differences.

### Structural context dictates whether circularization preserves dynamics

**Figure 3.**
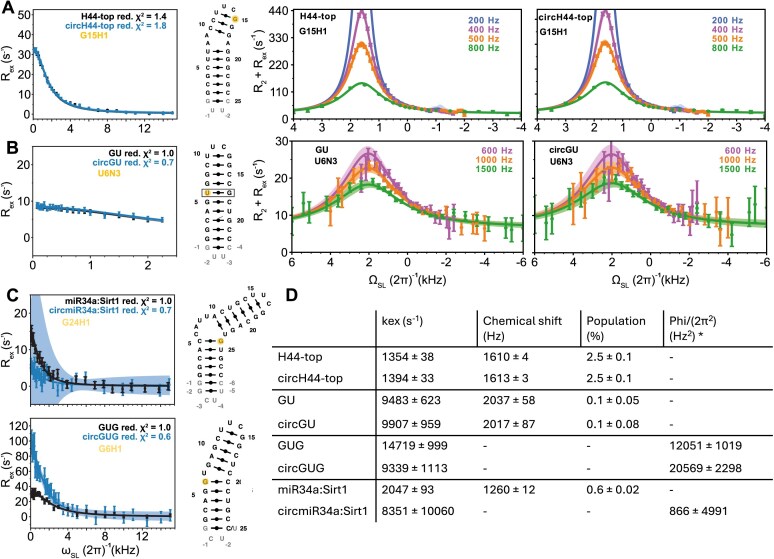
Comparison dynamics of linear and circRNAs using R_1ρ_ RD NMR. (**A**) On- and off-resonance profiles for G15H1 (yellow) of H44-top, and circH44-top measured at 25°C fit to equation 6 (Supporting Methods). (**B**) On- and off-resonance profiles for U6N3 (yellow) of GU and circGU at 25°C fit to equation 6 (Supporting Methods). (**C**) On-resonance overlay of linear and circular constructs, circMiR34a:Sirt1 G24H1 and circGUG G6H1 (indicated in yellow) samples measured at 25°C and 9°C, respectively. Data for MiR34a:Sirt1 on-resonance from Baronti *et al*. [6]. (**D**) Overview of fitted parameters for all constructs. For H44-top and GU tautomer the consistent fit parameters indicate that circularization does not influence dynamics, as visually identified by similar on- and off-resonance profiles. However, for MiR34a:Sirt1 G24H1 and GUG G6H1 clear differences are identified, even after further increasing the distance of the added tetraloop to the dynamic region (Supplementary Fig. S15). Circularization preserves native dynamics in H44-top and GU, but alters them in constructs whose excited states involve interhelical bending (MiR34a:Sirt1, GUG), revealing that A-form helices can cooperatively transmit dynamic information over long distances.

To quantitatively compare dynamics between the previously characterized linear and circularized constructs, we performed ^1^H and ^15^N R_1ρ_ relaxation dispersion (RD) NMR [52, 53, 70] (Figs 2 and 3A–C) on four diverse RNAs: (i) G15 in H44-top undergoes a base flipping from *syn* to *anti* coupled to a register shift that alters the base-pairing partner from U to C, as the tetraloop rearranges to a tri-loop (Fig. 3A) [58]; (ii) U6 in the GU construct tautomerizes from a GU wobble base pair to a WC-like GU mispair [10]; (iii) G24 in MiR34a:Sirt1 which switches the base-pairing partner from C to a U, structurally extending the seed (Fig. 2E) [6] and functionally increasing repression efficiency; and (iv) G6 in the GUG bulge model that reports on the dynamics of the single bulged U7 [52].

To monitor the impact of the tetraloop on the structure and dynamics, CSPs of the hairpins were analysed with respect to the dynamic region of interest (Supplementary Fig. S13). For circH44-top, circMir34a:Sirt1 and circGU, CSP were observed for up to three base pairs away from the altered region, as expected for structural perturbations [42, 71]. The dynamic nucleotides of all constructs lie outside of the chemical shift alterations (Supplementary Fig. S13). Interestingly, circGUG G5 to G8 and U18 all exhibit small CSP, despite being four or more base pairs away from the site of sequence alteration/added tetraloop, indicating a potential alteration of dynamics for this construct. In line with their unperturbed secondary structure, the circularization of H44-top and GU-tautomer preserved their original dynamics, maintaining their exchange rates, populations, and chemical shift of the excited state (Fig. 3A, B, and D), supported by overlapping on-resonance profiles and equivalent fit parameters (Fig. 3D).

In contrast, circularization of MiR34a:Sirt1 with a rigid tetraloop altered the dynamic extension of the seed, as indicated by the flattening of the on-resonance relaxation dispersion curve (Fig. 3C). MiR34a:Sirt1 contains two helices, connected by a four-nucleotide bulge, which, during the transition from GS to ES, undergo a large structural rearrangement with an altered helical bending angle [6]. Additionally, the dynamic process in GUG is also altered by the addition of the terminal tetraloop. CircGUG experiences a 40% reduction in the exchange rate (from *k*_ex,lin_ = 14718.63 ± 998.61 s^-1^ to *k*_ex,circ_ = 9339.01 ± 1112.87 s^-1^, Fig. 3D). This is consistent with the CSP in circGUG, which can be caused by altered chemical exchange. This dynamic alteration of circGUG is also indicated by a broadening of the G6 imino proton (Fig. 2F, blue) and an increased on-resonance curve (Fig. 3C). Due to the fast exchange of GUG and the reduced exchange (R_EX_) contribution of circMiR34a:Sirt1, additional exchange parameters could not be determined.

To evaluate the effect of circularization on GUG dynamics, the lower helix was extended by 3 base pairs and the tetraloop was replaced with the CUUG tetraloop, which was present in the constructs with preserved dynamics (GU and H44-top). However, for circGUGextended, the broadened G6 imino proton persisted, indicating that the dynamics are still altered despite being now eight base pairs away from the added tetraloop (Supplementary Fig. S15). The extension has increased the distance between the structural perturbation and the local dynamics, as demonstrated now with the absence of CSP for U22, indicating that the immediate structural impact of the mutation does not extend to the dynamic bulge. However, the CSPs of G5 and G8, flanking the bulge, are large and remain the same as for circGUG, confirming that the dynamic impact is longer range, and that the helix acts as a transporter of dynamic information. To evaluate the impact of the closing loop, circGUG was tested with the weakest tetraloop AACA [62] and a disrupted ACACA pentaloop, still resulting in a broadened G6 imino proton (Supplementary Fig. S16). These observations, alongside the reduced circMiR-34a:Sirt1 dynamics, suggest that the transition to a larger interhelical bend induced by local bulge dynamics or larger bulge dynamics require an unrestrained helix to accommodate the structural changes. Constraining the helix through circularization alters this dynamic process, indicating that RNA helices can cooperatively transmit information of local structural dynamics over several base pairs, consistent with their known bend persistence length, and reminiscent of allostery in proteins [72, 73].

### Circular RNA facilitates relaxation measurements in-cell

**Figure 4.**
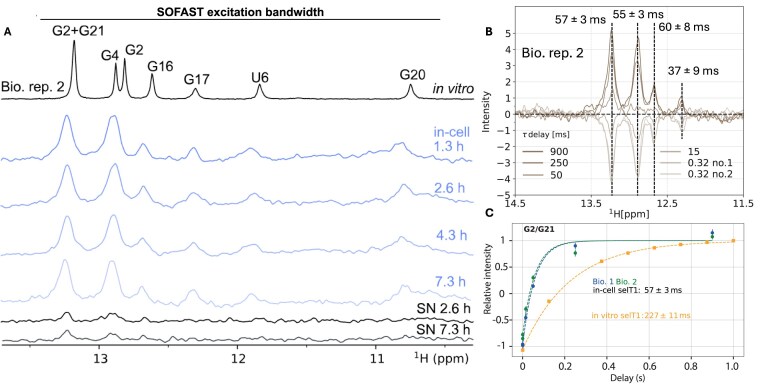
In-cell NMR of circular H44-top. (**A**) ^1^H 1D SOFAST in-cell time course of circH44-top for biological replicate 2 (blue, replicate 1 in Supplementary Fig. S10) compared to the *in vitro* reference spectrum (black). Each spectrum was acquired with 4096 scans with a D1 of 0.1 s, total time per spectra was 15 min and 21 s. Signal reduction was globally fitted between biological replicates to a linear decay model (Supplementary Fig. S11). The supernatant controls (SN) were performed according to the interstitial supernatant protocol (Supporting Methods). (**B**) In-cell selective inversion recovery experiment to determine the imino proton longitudinal relaxation time (selT_1_) of replicate 2 (replicate 1, Supplementary Fig. S10). Acquired with 512 scans, and between 19 and 32 min per experiment for the shortest and longest delay, respectively. (**C**) Exemplary fits for G2/G21H1 imino proton relaxation time in-cell and *in vitro* (fit to mono-exponential recovery, Materials and methods, other fits Supplementary Figs S17 and S18). Circularization enables, for the first time, relaxation measurements on chemically unmodified RNA in human cells. Specifically, the imino selT₁ is measured with intracellular relaxation accelerated ∼3–4-fold relative to *in vitro* allowing ∼3.4-fold faster acquisition (Supplementary Fig. S19).

NMR relaxation measurements report on dynamic properties of biomolecules, for instance, selT_1_ acts as a reporter on molecular flexibility, tumbling, base pair-opening, and paramagnetic species [33, 57, 74, 75]. The extended lifetime of circH44-top allowed for longer in-cell measurements, rendering it feasible to record selective ^1^H imino longitudinal relaxation time (selT_1_) inside human cells of unmodified RNA for the first time. Interleaved selective inversion recovery experiments were performed on each of the two biological replicates. The measured selT_1_ of RNA in-cell (G2/G21 57 ± 3 ms, G4/G23 55 ± 3 ms, G16 60 ± 8 ms, and G17 37 ± 9 ms) were reproducible, and were globally fitted, (Fig. 4, and Supplementary Figs S10 and S17). Comparing to *in vitro* selT_1_ (G2/G21 227 ± 11 ms, G4 204 ± 10 ms, G23 173 ± 9 ms, G16 241 ± 12 ms, and G17 108 ± 5 ms) a significant increase of the relaxation rate inside the cell was observed, ranging from 2.9- to 4-fold larger (Fig. 4C, and Supplementary Figs S10 and S17), consistent with the previous study on DNA, which showed a change of 2.8–7.2. As previously shown for DNA, the application of the optimized recovery delays (10 ms) for the SOFAST experiment resulted in an 88% increase in integrated peak volume compared to spectra with unoptimized short recovery delay (100 ms) (Supplementary Fig. S19). Correspondingly, the same signal to noise can be achieved 3.4× faster (here 4 min and 30 s as opposed to 15 min and 21 s), facilitating more complex experiments.

## Discussion

Transient RNA structural dynamics play a central role in regulating cellular functions and are sensitive to the cellular environment [5, 9, 10, 12, 15, 76]. In-cell NMR seeks to quantitatively measure RNA dynamics in their native context but is limited by rapid intracellular degradation of unmodified RNA [5, 6, 9, 10, 12, 14, 15, 17–19, 72, 77, 78].

### A general and scalable platform for circular RNA production

Here, we apply the circularization method developed by Chen *et al*. [40] to the NMR construct design of hairpin motifs [15, 42] often employed for NMR studies [10, 41–44]). We establish a modular and scalable enzymatic pipeline for circularizing RNA hairpins that is compatible with multiple production methods, including tandem IVT [49], conventional IVT [64, 67], and solid-phase synthesis [79] (Figs 1 and 2, and Supplementary Fig. S2). By exploiting the purity of RNA produced by tandem IVT, high efficiency of ligation (65%–96%) was achieved. Utilizing the dPAGE band migration change associated with circRNA, highly pure circRNA was produced at large scale (1200 nmol) (Fig. 1A and Supplementary Fig. S2) [40, 49, 65]. Alternative production methods, such as 5′-*cis*-acting ribozymes [66] can also be utilized, if the pre-requisite 5′-monophosphate is incorporated before ligation. The circularization is unlikely to be of use for RNAs where the function requires accessible or asymmetric 5′- and 3′-terminal ends, e.g. exonuclease resistant RNA motif, or dynamic RNAs where ends exchange in length [76, 80]. Enzymatic and efficient pipelines are particularly critical for in-cell NMR, where large quantities of RNA are required and provide a practical route toward isotopically labelled circRNA without the cost and limitations of chemical synthesis.

### Circularization stabilizes RNA while preserving native structure

Increased stability of RNA is necessary for in-cell NMR, to avoid degradation by cellular exonucleases, which limits experimental time. Circularization dramatically enhances RNA stability, extending lifetimes in cell lysate from 1 h, for linear RNA, to over 24 h, comparable to fully 2′-*O*-methylated RNA (Fig. 1C and Supplementary Fig. S3) [27, 81, 82]. Under in-cell conditions at 25°C, Broft *et al*. demonstrate a signal half-life of approximately 1–2 h for a linear hairpin in HeLa cells, whilst here, also at 25°C, we observe minimal reduction of circH44-top NMR signal over 7 h (Supplementary Fig. S11), with lysate stability assay indicating a half-life of >24 h (Supplementary Fig. S3) [27]. The apparent signal reduction determined in-cell relies on signal intensities, which can decay not only due to degradation but also larger complex formation inside the cell. While in-cell NMR experiments are often conducted at below physiological temperature to prolong cell viability and minimize RNA degradation [83], this likely impacts cellular metabolism and the cellular milieu as a result.

Additionally, stabilization should not disrupt the native structure, which is often a limitation for certain modifications, i.e. 2′-*O*-methylation which can alter sugar puckering, tetraloop formation and intermolecular interactions, as observed in methoxy H44-top (Fig. 3A and Supplementary Fig. S14) [36–38, 84, 85] and also discussed in Hänsel *et al*. [28]. Whereas circularization preserved the secondary structure for all constructs (Fig. 2 and Supplementary Fig. S12).

### Circularization reveals dynamic cooperativity via A-form helices

Despite RNA structural equilibria being highly sensitive to molecular interactions, circularization preserved dynamics for both H44-top and the GU tautomerization (Fig. 3A and B), indicating this is a valuable method of stabilization. MiR34a:Sirt1 and GUG dynamics were altered upon circularization, while their secondary structure was maintained (Fig. 2 and Supplementary Fig. S12). MiR34a:Sirt1 undergoes a seed extension, which leads to a change in location of the bulge and large alteration of the angle between the two helices in the excited state [6], while the uridine bulge in GUG switches between stacking intrahelically and flipping out of the helix, likely altering the angle between the two helices [52]. As dynamics were altered in both constructs that undergo changes in helical bend angle, we hypothesize that these changes in helical bend angle require the flexibility of either, or both, the upper and lower helices as compensatory rearrangements of the otherwise rigid A-form helix, presenting a transduction of dynamic signal, reminiscent of protein allostery. This rearrangement, however, is not compatible with the addition of a rigid UUCG, or CUUG, or less stable AACA tetraloop or ACACA pentaloop and therefore leads to an alteration of the dynamic properties of these systems. Extending the length of the helix from where the tetraloop is added, circGUGextended or altering the loop sequence (Supplementary Fig. S15), does not prevent alteration of the dynamics, indicating that the flexibility requirements are longer range. The circularization presents an artificial restraint, and therefore is not reflecting an actual allosteric system; however, it reveals the possible mechanism for cooperative behaviour and sheds light on the role of typically non-dynamic helices in RNA structures as transferring dynamics over larger distances, allowing for allostery. Together this indicates a cooperative mechanism of RNA dynamics, in which the structural impact of local dynamics are transferred over larger distances through the helix. These observations are consistent with measurements of RNA bend persistence length of up to 200 bp [73, 86], and previous work identifying correlated motion of RNA helices connected by a flexible region, although the exact mechanism remained unclear [87]. Additionally, the use of less stable loops, namely AACA and ACACA to circularize GUG were also unable to recapitulate the original dynamics of linear GUG. Future studies could more extensively explore the use of more flexible terminal loops or pseudo-triloops for circularization of RNA hairpins undergoing changes in the helical bend during dynamic rearrangement, to preserve the dynamics of the original sequence, or alternatively use tetraloops to probe longer distance cooperativity of RNA helices.

### Enhanced lifetime enables in-cell measurements of RNA dynamics

Following electroporation of circH44-top into HeLa cells, in-cell imino selective inversion recovery experiments (selT_1_) reveal 2.9-4 fold faster imino longitudinal relaxation rates (selR_1_) inside the cell (Fig. 4 and Supplementary Fig. S19), in agreement with in-cell imino proton relaxation rates of a DNA duplex [33]. These relaxation rates can be affected by a variety of factors, including reduced molecular tumbling, paramagnetic species, and altered base-pair opening and/or proton exchange in the intracellular environment [33, 57, 74], and it remains to be determined which of these play a significant role. To remove the confounding contribution of cellular respiration to pH, and other cellular pathways, in-cell experiments would ideally be performed using a bioreactor to supply cells with fresh media. Careful *in vitro* reconstitution experiments will be required to delineate the differing effects of intracellular crowding, pH, ionic strength, and paramagnetic species on the selT_1_. The reduced selT_1_ enables a signal enhancement of 88% in-cell with an optimized shorter D1 time, facilitating faster measurements for future in-cell NMR of RNA, important for example, in in-cell protein- [48, 62], or ligand–RNA binding real-time NMR.

In this work, we establish a readily adaptable pipeline to design and produce circRNA, which preserves the structure of the native RNA for all sequences tested, and dynamics of the GU and H44-top sequences. Interestingly, circularizing RNAs containing larger helical bending re-arrangements (MiR34a:Sirt1 and GUG) alters their exchange, showing that the influence of local dynamics can be relayed over the RNA helix. The circularization significantly extends the lifetime of the RNA due to the resistance to exonuclease activity, enabling the first reproducible and precise relaxation measurements in-cell on chemically unmodified RNA, and promises extended in-cell NMR studies of RNA dynamics by future combination with bioreactors. This opens the way for future applications, including probing RNA interactions with proteins, small molecules, or other nucleic acids in a cellular environment as well as studying conformational switching and allosteric regulation in a biologically relevant context.

## Supplementary Material

gkag675_Supplemental_File

## Data Availability

All data are present in the manuscript and raw data, alongside relaxation dispersion analysis scripts, are available at https://doi.org/10.5281/zenodo.20748774.

## Notes

Present address: Institute for Organic Chemistry and Chemical Biology, Center of Biomolecular Magnetic Resonance (BMRZ), Goethe University Frankfurt am Main, 60438 Germany

## References

[1] Spitale RC, Incarnato D. Probing the dynamic RNA structurome and its functions. Nat Rev Genet. 2023;24:178–96. 10.1038/s41576-022-00546-w. 36348050 PMC9644009

[2] Corbett AH . Post-transcriptional regulation of gene expression and human disease. Curr Opin Cell Biol. 2018;52:96–104. 10.1016/j.ceb.2018.02.011. 29518673 PMC5988930

[3] Dykes IM, Emanueli C. Transcriptional and post-transcriptional gene regulation by long non-coding RNA. Genomics Proteomics Bioinformatics. 2017;15:177–86. 10.1016/j.gpb.2016.12.005. 28529100 PMC5487525

[4] Reinhart BJ, Slack FJ, Basson M et al. The 21-nucleotide let-7 RNA regulates developmental timing in *Caenorhabditis elegans*. Nature. 2000;403:901–6. 10.1038/35002607. 10706289

[5] Baisden JT, Boyer JA, Zhao B et al. Visualizing a protonated RNA state that modulates microRNA-21 maturation. Nat Chem Biol. 2021;17:80–8. 10.1038/s41589-020-00667-5. 33106660

[6] Baronti L, Guzzetti I, Ebrahimi P et al. Base-pair conformational switch modulates miR-34a targeting of Sirt1 mRNA. Nature. 2020;583:139–44. 10.1038/s41586-020-2336-3. 32461691

[7] Zeng X, Chugh J, Casiano-Negroni A et al. Flipping of the ribosomal A-site adenines provides a basis for tRNA selection. J Mol Biol. 2014;426:3201–13. 10.1016/j.jmb.2014.04.029. 24813122 PMC4150856

[8] Ganser LR, Chu CC, Bogerd HP et al. Probing RNA conformational equilibria within the functional cellular context. Cell Rep. 2020;30:2472–80. 10.1016/j.celrep.2020.02.004. 32101729 PMC7941409

[9] Mustoe AM, Brooks CL, HM Al-Hashimi. Hierarchy of RNA functional dynamics. Annu Rev Biochem. 2014;83:441–66. 10.1146/annurev-biochem-060713-035524. 24606137 PMC4048628

[10] Dethoff EA, Petzold K, Chugh J et al. Visualizing transient low-populated structures of RNA. Nature. 2012;491:724–8. 10.1038/nature11498. 23041928 PMC3590852

[11] Alvey HS, Gottardo FL, Nikolova EN et al. Widespread transient Hoogsteen base pairs in canonical duplex DNA with variable energetics. Nat Commun. 2014;5. 10.1038/ncomms5786. PMC4537320 25185517

[12] Kimsey IJ, Petzold K, Sathyamoorthy B et al. Visualizing transient Watson-Crick-like mispairs in DNA and RNA duplexes. Nature. 2015;519:315–20. 10.1038/nature14227. 25762137 PMC4547696

[13] Marušič M, Schlagnitweit J, Petzold K. RNA dynamics by NMR spectroscopy. ChemBioChem. 2019;20:2685–710. 10.1002/cbic.201900072. 30997719 PMC6899578

[14] Rangadurai A, Szymaski ES, Kimsey IJ et al. Characterizing micro-to-millisecond chemical exchange in nucleic acids using off-resonance R1ρ relaxation dispersion. Prog Nucl Magn Reson Spectrosc. 2019;112-113:55–102. 10.1016/j.pnmrs.2019.05.002. 31481159 PMC6727989

[15] HM Al-Hashimi . NMR studies of nucleic acid dynamics. J Magn Reson. 2013;237:191–204. 10.1016/j.jmr.2013.08.014. 24149218 PMC3984477

[16] Alderson TR, Kay LE. NMR spectroscopy captures the essential role of dynamics in regulating biomolecular function. Cell. 2021;184:577–95. 10.1016/j.cell.2020.12.034. 33545034

[17] Tyrrell J, Weeks KM, Pielak GJ. Challenge of mimicking the influences of the cellular environment on RNA structure by PEG-induced macromolecular crowding. Biochemistry. 2015;54:6447–53. 10.1021/acs.biochem.5b00767. 26430778 PMC4893815

[18] Tyrrell J, McGinnis JL, Weeks KM et al. The cellular environment stabilizes adenine riboswitch RNA structure. Biochemistry. 2013;52:8777–85. 10.1021/bi401207q. 24215455 PMC4905562

[19] Kim YB, Wacker A, von Laer K et al. Ligand binding to 2-deoxyguanosine sensing riboswitch in metabolic context. Nucleic Acids Res. 2017;45:gkx016. 10.1093/nar/gkx016. PMC5435998 28115631

[20] Broft P, Dzatko S, Krafcikova M et al. In-cell NMR spectroscopy of functional riboswitch aptamers in eukaryotic cells. Angew Chem Int Ed. 2021;60:865–72. 10.1002/anie.202007184. PMC7839747 32975353

[21] Bao HL, Xu Y. Telomeric DNA–RNA–hybrid G-quadruplex exists in environmental conditions of HeLa cells. Chem Commun. 2020;56:6547–50. 10.1039/D0CC02053B. 32396161

[22] Yamaoki Y, Nagata T, Kondo K et al. Shedding light on the base-pair opening dynamics of nucleic acids in living human cells. Nat Commun. 2022;13. 10.1038/s41467-022-34822-4. PMC9708698 36446768

[23] Bao HL, Masuzawa T, Oyoshi T et al. Oligonucleotides DNA containing 8-trifluoromethyl-2’-deoxyguanosine for observing Z-DNA structure. Nucleic Acids Res. 2020;48:7041–51. 10.1093/nar/gkaa505. 32678885 PMC7367190

[24] Sakamoto T, Yamaoki Y, Nagata T et al. Detection of parallel and antiparallel DNA triplex structures in living human cells using in-cell NMR. Chem. Commun. 2021;57:6364–7. 10.1039/D1CC01761F. 34137388

[25] Yamaoki Y, Kiyoishi A, Miyake M et al. The first successful observation of in-cell NMR signals of DNA and RNA in living human cells. Phys Chem Chem Phys. 2018;20:2982–5. 10.1039/C7CP05188C. 29022027

[26] Schlagnitweit J, Friebe Sandoz S, Jaworski A et al. Observing an antisense drug complex in intact Human cells by in-cell NMR spectroscopy. ChemBioChem. 2019;20:2474–8. 10.1002/cbic.201900297. 31206961

[27] Broft P, Dzatko S, Krafcikova M et al. In-cell NMR spectroscopy of functional riboswitch aptamers in eukaryotic cells. Angew Chem. 2021;133:878–85. 10.1002/ange.202007184. PMC7839747 32975353

[28] Hänsel R, Foldynová-Trantírková S, Löhr F et al. Evaluation of parameters critical for observing nucleic acids inside living *Xenopus laevis* oocytes by in-cell NMR spectroscopy. J Am Chem Soc. 2009;131:15761–8. 10.1021/ja9052027. 19824671

[29] Bao HL, Xu Y. Telomeric DNA–RNA–hybrid G-quadruplex exists in environmental conditions of HeLa cells. Chem Commun. 2020;56:6547–50. 10.1039/D0CC02053B. 32396161

[30] Barbieri L, Luchinat E. Monitoring protein–ligand interactions in human cells by real-time quantitative in-cell nmr using a high cell density bioreactor. J Vis Exp. 2021;2021. 10.3791/62323. 33779617

[31] Rynes J, Istvankova E, Dzurov Krafcikova M et al. Protein structure and interactions elucidated with in-cell NMR for different cell cycle phases and in 3D human tissue models. Commun Biol. 2025;8. 10.1038/s42003-025-07607-w. PMC11806009 39920376

[32] Giassa IC, Rynes J, Fessl T et al. Advances in the cellular structural biology of nucleic acids. FEBS Lett. 2018;592:1997–2011. 10.1002/1873-3468.13054. 29679394

[33] Annecke HTP, Eidelpes R, Feyrer H et al. Optimising in-cell NMR acquisition for nucleic acids. J Biomol NMR. 2024;78:249–64. 10.1007/s10858-024-00448-5. 39162911 PMC11614993

[34] Ghosh R, Xiao Y, Kragelj J et al. In-cell sensitivity-enhanced NMR of intact viable mammalian cells. J Am Chem Soc. 2021;143:18454–66. 10.1021/jacs.1c06680. 34724614 PMC8961430

[35] Hyjek-Składanowska M, Vickers TA, Napiórkowska A et al. Origins of the increased affinity of phosphorothioate-modified therapeutic nucleic acids for proteins. J Am Chem Soc. 2020;142:7456–68. 10.1021/jacs.9b13524. 32202774

[36] Adamiak DA, Milecki J, Popenda M et al. Crystal structure of 2′-*O*-Me(CGCGCG)2, an RNA duplex at 1.30 Å resolution. Hydration pattern of 2′-*O*-methylated RNA. Nucleic Acids Res. 1997;25:4599–607. 10.1093/nar/25.22.4599. 9358171 PMC147083

[37] Abou Assi H, Rangadurai AK, Shi H et al. 2′-*O*-Methylation can increase the abundance and lifetime of alternative RNA conformational states. Nucleic Acids Res. 2021;48:12365–79. 10.1093/nar/gkaa928. PMC7708057 33104789

[38] Khoshnevis S, Dreggors-Walker RE, Marchand V et al. Ribosomal RNA 2′-*O*-methylations regulate translation by impacting ribosome dynamics. Proc Natl Acad Sci USA. 2022;119. 10.1073/pnas.2117334119. PMC8944910 35294285

[39] Abe N, Abe H, Nagai C et al. Synthesis, structure, and biological activity of dumbbell-shaped nanocircular RNAs for RNA interference. Bioconjug Chem. 2011;22:2082–92. 10.1021/bc2003154. 21899349

[40] Chen H, Cheng K, Liu X et al. Preferential production of RNA rings by T4 RNA ligase 2 without any splint through rational design of precursor strand. Nucleic Acids Res. 2020;48:e54. 10.1093/nar/gkaa181. 32232357 PMC7229815

[41] Wacker A, Weigand JE, Akabayov SR et al. Secondary structure determination of conserved SARS-CoV-2 RNA elements by NMR spectroscopy. Nucleic Acids Res. 2020; 48:12415–35. 10.1093/nar/gkaa1013. 33167030 PMC7736788

[42] Riad M, Hopkin N, Baronti L et al. Mutate-and-chemical-shift-fingerprint (MCSF) to characterize excited states in RNA using NMR spectroscopy. Nat Protoc. 2021;16:5146–70. 10.1038/s41596-021-00606-1. 34608336

[43] Petzold K, Duchardt E, Flodell S et al. Conserved nucleotides in an RNA essential for hepatitis B virus replication show distinct mobility patterns. Nucleic Acids Res. 2007;35:6854–61. 10.1093/nar/gkm774. 17933777 PMC2175316

[44] Han G, Xue Y. Rational design of hairpin RNA excited states reveals multi-step transitions. Nat Commun. 2022;13. 10.1038/s41467-022-29194-8. PMC8938425 35314698

[45] Abe N, Abe H, Ito Y. Dumbbell-shaped nanocircular RNAs for RNA interference. J Am Chem Soc. 2007;129:15108–9. 10.1021/ja0754453. 18001025

[46] Jahns H, Degaonkar R, Podbevsek P et al. Small circular interfering RNAs (sciRNAs) as a potent therapeutic platform for gene-silencing. Nucleic Acids Res. 2021;49:10250–64. 10.1093/nar/gkab724. 34508350 PMC8501968

[47] Terrazas M, Ivani I, Villegas N et al. Rational design of novel *N*-alkyl-N capped biostable RNA nanostructures for efficient long-term inhibition of gene expression. Nucleic Acids Res. 2016;44:4354–67. 10.1093/nar/gkw169. 26975656 PMC4872095

[48] Y.-x.ma M, Mccallum K, Climie SC et al. Design and synthesis of RNA miniduplexes via a synthetic linker approach. 2. Generation of covalently closed, double-stranded cyclic HIV-1 TAR RNA analogs with high tat-binding affinity. Nucl Acids Res. 1993;21:2585–9. 10.1093/nar/21.11.2585. 8332456 PMC309585

[49] Feyrer H, Munteanu R, Baronti L et al. One-pot production of RNA in high yield and purity through cleaving tandem transcripts. Molecules. 2020;25:1142. 10.3390/molecules25051142. 32143353 PMC7179201

[50] Toews S, Donà F, Keller M et al. Targeting the SARS-CoV-2 RNA translation initiation element SL1 by molecules of low molecular weight. J Am Chem Soc. 2025;147:28783–98. 10.1021/jacs.5c05264. 40758647 PMC12356538

[51] Viskova P, Krafcik D, Trantirek L et al. In-cell NMR spectroscopy of nucleic acids in Human cells. CP Nucleic Acid Chem. 2019;76. 10.1002/cpnc.71. 30489693

[52] Schlagnitweit J, Steiner E, Karlsson H et al. Efficient detection of structure and dynamics in unlabeled RNAs: the SELOPE approach. Chem A Eur J. 2018;24:6067–70. 10.1002/chem.201800992. PMC5947647 29504639

[53] Steiner E, Schlagnitweit J, Lundström P et al. Capturing excited states in the fast-intermediate exchange limit in biological systems using 1 H NMR spectroscopy. Angew Chem. 2016;128:16101–4. 10.1002/ange.201609102. 27860024

[54] Palmer AG, Kroenke CD, Loria JP. Nuclear magnetic resonance methods for quantifying microsecond-to-millisecond motions in biological macromolecules. Methods Enzymol. 2001;10:204–38. 10.1016/S0076-6879(01)39315-1. 11462813

[55] Miloushev VZ, Palmer AG. R1ρ relaxation for two-site chemical exchange: general approximations and some exact solutions. J Magn Reson. 2005;177:221–7. 10.1016/j.jmr.2005.07.023. 16143548

[56] Geen H, Freeman R. Band-selective radiofrequency pulses. J Magn Res. 1991;93:93–141. 10.1016/0022-2364(91)90034-Q.

[57] De L’universite D, Fourier J. Universite Joseph Fourier-grenoble 1 Ecole Doctorale de Physique these pour obtenir Le grade de Paul schanda développement et applications de méthodes RMN rapides pour l’étude de la structure et de la dynamique des protéines development and application of fast NMR methods for the study of protein structure and dynamics.

[58] Steinmetzger Christian, Karlsson Hampus, Fontana Carolina et al. Universal 3D motif dynamics in RNA: the A-minor switch. EMPIAR dataset. 2026; bioRxiv. 10.64898/2026.05.08.718354.

[59] Groebe DR, Uhlenbeck OC. Characterization of RNA hairpin loop stability. Nucleic Acids Res. 1988;16:11725–35. 10.1093/nar/16.24.11725. 3211748 PMC339106

[60] Kuznetsov S V., Ren CC, Woodson SA et al. Loop dependence of the stability and dynamics of nucleic acid hairpins. Nucleic Acids Res. 2008;36:1098–112. 10.1093/nar/gkm1083. 18096625 PMC2275088

[61] Rentzeperis D, Alessi K, Marky LA. Thermodynamics of DNA hairpins: contribution of loop size to hairpin stability and ethidium binding. Nucleic Acids Res. 1993;21:2683–9. 10.1093/nar/21.11.2683. 8332464 PMC309599

[62] Sheehy JP, Davis AR, Znosko BM. Thermodynamic characterization of naturally occurring RNA tetraloops. RNA. 2010;16:417–29. 10.1261/rna.1773110. 20047989 PMC2811670

[63] Proctor DJ, Schaak JE, Bevilacqua JM et al. Isolation and characterization of a family of stable RNA tetraloops with the motif YNMG that participate in tertiary interactions. Biochemistry. 2002;41:12062–75. 10.1021/bi026201s. 12356306

[64] Karlsson H, Baronti L, Petzold K. A robust and versatile method for production and purification of large-scale RNA samples for structural biology. RNA. 2020;26:1023–37. 10.1261/rna.075697.120. 32354720 PMC7373988

[65] Tabak HF, van der Horst G, Smit J et al. Discrimination between RNA circles, interlocked RNA circles and lariats using two-dimensional polyacrylamide gel electrophoresis. Nucleic Acids Res. 1988;16:6597–605. 10.1093/nar/16.14.6597. 2456529 PMC338316

[66] Mörl M, Lizano E, Willkomm DK et al. Production of RNAs with homogeneous 5′ and 3′ Ends. In: Handbook of RNA Biochemistry. Wiley-VCH Verlag GmbH & Co.KGaA, Weinheim, Germany, 2008. 10.1002/9783527619504.ch2.

[67] Schnieders R, Knezic B, Zetzsche H et al. NMR spectroscopy of large functional RNAs: from sample preparation to low-gamma detection. CP Nucleic Acid Chem. 2020;82. 10.1002/cpnc.116. 32960489

[68] Krafcikova M, Dzatko S, Caron C et al. Monitoring DNA–ligand interactions in living Human cells using NMR spectroscopy. J Am Chem Soc. 2019;141:13281–5. 10.1021/jacs.9b03031. 31394899

[69] Tuma RS, Beaudet MP, Jin X et al. Characterization of SYBR gold nucleic acid gel stain: a dye optimized for use with 300-nm ultraviolet transilluminators. Anal Biochem. 1999;268:278–88. 10.1006/abio.1998.3067. 10075818

[70] Korzhnev DM, Orekhov VY, Kay LE. Off-resonance R1ρ NMR studies of exchange dynamics in proteins with low spin-lock fields: an application to a fyn SH3 domain. J Am Chem Soc. 2005;127:713–21. 10.1021/ja0446855. 15643897

[71] Shi H, Clay MC, Rangadurai A et al. Atomic structures of excited state A–T Hoogsteen base pairs in duplex DNA by combining NMR relaxation dispersion, mutagenesis, and chemical shift calculations. J Biomol NMR. 2018;70:229–44. 10.1007/s10858-018-0177-2. 29675775 PMC6048961

[72] Duchardt-Ferner E, Gottstein-Schmidtke SR, Weigand JE et al. What a difference an OH makes: conformational dynamics as the basis for the ligand specificity of the neomycin-sensing riboswitch. Angew Chem Int Ed. 2016;55:1527–30. 10.1002/anie.201507365. 26661511

[73] Dong HL, Zhang C, Dai L et al. The origin of different bending stiffness between double-stranded RNA and DNA revealed by magnetic tw eez ers and simulations. Nucleic Acids Res. 2024;52:2519–29. 10.1093/nar/gkae063. 38321947 PMC10954459

[74] Szulik MW, Voehler M, Stone MP. NMR analysis of base-pair opening kinetics in DNA. CP Nucleic Acid Chem. 2014;59. 10.1002/0471142700.nc0720s59. PMC4274747 25501592

[75] Guéron M, Leroy JL. Studies of base pair kinetics by NMR measurement of proton exchange. Methods Enzymol. 1995;261:383–13. 10.1016/S0076-6879(95)61018-9. 8569504

[76] Thompson RD, Carbaugh DL, Nielsen JR et al. Lifetime of ground conformational state determines the activity of structured RNA. Nat Chem Biol. 2025;21:1021–9. 10.1038/s41589-025-01843-1. 39939412 PMC12202162

[77] Dzatko S, Fiala R, Hänsel-Hertsch R et al. In-cell NMR spectroscopy of nucleic acids. New developments in NMR. 2020.

[78] Dzatko S, Krafcikova M, Hänsel-Hertsch R et al. Evaluation of the stability of DNA i-motifs in the nuclei of living mammalian cells. Angew Chem Int Ed. 2018;57:2165–9. 10.1002/anie.201712284. PMC5820743 29266664

[79] Marshall WS, Kaiser RJ. Recent advances in the high-speed solid phase synthesis of RNA. Curr Opin Chem Biol. 2004;8:222–9. 10.1016/j.cbpa.2004.04.012. 15183319

[80] Fürtig B, Wenter P, Reymond L et al. Conformational dynamics of bistable RNAs studied by time-resolved NMR spectroscopy. J Am Chem Soc. 2007;129:16222–9. 10.1021/ja076739r. 18047344

[81] Allerson CR, Sioufi N, Jarres R et al. Fully 2′-modified oligonucleotide duplexes with improved *in vitro* potency and stability compared to unmodified small interfering RNA. J Med Chem. 2005;48:901–4. 10.1021/jm049167j. 15715458

[82] Czauderna F, Fechtner M, Dames S et al. Structural variations and stabilising modifications of synthetic siRNAs in mammalian cells. Nucleic Acids Res. 2003;31:2705–16. 10.1093/nar/gkg393. 12771196 PMC156727

[83] Foldynova-Trantirkova S, Harnos J, Rynes J et al. In-cell NMR spectroscopy of nucleic acids: basic concepts, practical aspects, and applications. Prog Nucl Magn Reson Spectrosc. 2025;148-149:101560. 10.1016/j.pnmrs.2025.101560. 40912878

[84] Williams DJ, Hall KB. Experimental and theoretical studies of the effects of deoxyribose substitutions on the stability of the UUCG tetraloop. J Mol Biol. 2000;297:251–65. 10.1006/jmbi.2000.3547. 10704320

[85] Williams DJ, Boots JL, Hall KB. Thermodynamics of 2′-ribose substitutions in UUCG tetraloops. RNA. 2001;7:44–53. 10.1017/S1355838201001558. 11214179 PMC1370067

[86] Herrero-Galán E, Fuentes-Perez ME, Carrasco C et al. Mechanical identities of RNA and DNA double helices unveiled at the single-molecule level. J. Am. Chem. Soc. 2013;135:122–31. 10.1021/ja3054755. 23214411

[87] Zhang Q, Stelzer AC, Fisher CK et al. Visualizing spatially correlated dynamics that directs RNA conformational transitions. Nature. 2007;450:1263–7. 10.1038/nature06389. 18097416

