# Supplementary material for: RNA circularization preserves dynamics and enables in-cell relaxation NMR
Source: Nucleic Acids Res. 2026 Jul 14;54(13):gkag675. doi: 10.1093/nar/gkag675 (PMC13365984; doi:10.1093/nar/gkag675)
Supplement: gkag675_Supplemental_File [file gkag675_supplemental_file.pdf]

Supporting Information

RNA Circularisation Preserves Dynamics and Enables In-cell Relaxation NMR

Authors: Henry T. P. Annecke[1,2]†, Katja Bekcic[1]†, Sabrina Toews[2,3], Hannes Feyrer[2], Katja Petzold[1,2]*

1. Department of Medical Biochemistry and Microbiology, Center of Excellence for the Chemical Mechanisms & Science for Life Laboratory, Uppsala University, Uppsala, Sweden
2. Department of Medical Biochemistry and Biophysics, Karolinska Institute, Stockholm, Sweden
3. current address: Institute for Organic Chemistry and Chemical Biology, Center of Biomolecular Magnetic Resonance (BMRZ), Goethe University Frankfurt am Main, Germany

† These authors contributed equally

* corresponding author, email: katja.petzold@imbim.uu.se

Supporting Materials and Methods

In vitro transcription (IVT)

Conventional *in vitro* transcription was carried out as in Karlsson *et al.* (1) to produce linear GU, circGU, and circmiR34a:Sirt1. First, the T7 promoter sequence (IDT, standard desalting) was annealed with template strand (Sigma-Aldrich, standard desalting) modified with two 5' terminal methoxy bases, in 40 mM Tris-HCl pH 8 (Sigma Aldrich, C4706), 1 mM EDTA pH 8 (Sigma-Aldrich E5134), and 150 mM NaCl (Sigma-Aldrich S3014) at 50 µM, by heating to 90 °C for 5 minutes followed by cooling to room temperature. Optimised IVT reaction conditions used for linear GU were 100 mM Tris-Glutamate pH 8 (Sigma-Aldrich 10708976001, 49449), 15 mM $Mg(OAc)_2$ (Sigma-Aldrich M5661), 7.5 mM Spermidine (Sigma-Aldrich S2501), 10% DMSO (Sigma-Aldrich 41639), 10 mM DTT (Sigma-Aldrich D0632), 4 mM GMP (Sigma-Aldrich G8377), 3 mM GTP (Thermofisher J61414.03), 3 mM UTP (Thermofisher 226310010), 3 mM CTP (J62238.03), 3 mM ATP (Sigma-Aldrich A2383), 2 µM dsDNA template Supplementary Table 4, and 0.46 mg/mL T7 polymerase P266L (Protein Science Facility, Scilifelab), in 7.5 mL final volume. For circGU 100 mM Tris-Glutamate pH 8, 10 mM $Mg(OAc)_2$, 5 mM Spermidine, 15 % DMSO, 20 mM DTT, 6 mM GMP, 3 mM GTP, UTP, CTP, and ATP, 2 µM dsDNA template, 0.46 mg/mL T7 polymerase P266L, in 10 mL total volume. For circmiR34a:Sirt1 100 mM Tris-Glutamate pH 8, 5 mM $Mg(OAc)_2$, 5 mM spermidine, 10% DMSO, 15 mM DTT, 6 mM GMP, 3 mM GTP, UTP, CTP, and ATP, 2 µM dsDNA template, 0.46 mg/mL T7 polymerase P266L, in 10 mL total volume. This was incubated at 37 °C. shaking at 300 rpm in 10 mL reaction volume for 16 hours. Reaction was quenched with 20% v/v 500 mM EDTA pH 8, and concentrated in 3 kDa Amicon centrifugal filters (Milli-pore) before gel purification.

Solid-phase synthesis

circGUG was prepared at 1 µmol scale by solid-phase synthesis on an H-8 DNA/RNA/LNA synthesizer (K&A Laborgeräte). Amidites Bz-A-CE, Ac-C-CE, iBu-G-CE, U-CE (Sigma-Aldrich) where dissolved in acetonitrile to 70 mM. Synthesis was performed DMT-OFF with 12 minute coupling times. The RNA was cleaved from columns using 1 mL AMA (14% ammonium hydroxide (Sigma-Aldrich 221228) with 20% aqueous methylamine (Sigma-Aldrich 426466)) for 30 min at room temperature and then washed with 0.5 mL AMA. Protecting groups were removed by incubation at 65 °C for 30 minutes. Samples were speedvac dried, (Savant SpeedVac, ThermoScientific), and then lyophilised overnight. The RNA was

dissolved in 100 µL anhydrous DMSO and 2′-hydroxyl protecting group were removed with 125 µL TEA.3HF at 65 °C for 2.5 hours. The RNA was ethanol precipitated and then purified by denaturing polyacrylamide gel electrophoresis (dPAGE).

Analytical PAGE

Analytical denaturing PAGE gels were prepared from a solution of 10-20% acrylamide:bis 19:1, 8M Urea and TBE buffer (100 mM Tris, 90 mM boric acid, 1 mM EDTA). Gels were cast in plates 0.75 mm apart with the addition of 4 µL TEMED and 40 µL 10% ammonium persulphate solution incubated for 15 minutes at RT. Gels were performed in 1x TBE using the Mini-PROTEAN Tetra System (Bio-Rad) at 350 V, following 15 minutes of pre-heating. Sample amounts of 0.2 – 2 pmol were loaded into 1 mm lanes, and 0.4 – 4 pmol into 2 mm lanes. Dye mix used was a 2 – 4x stock of 80% formamide, 50 mM EDTA and 380 µM bromphenol blue. Gels were stained with SYBR Gold and imaged on an iBright 750 (Invitrogen).

Preparative PAGE

Preparative denaturing PAGE gels were prepared from a solution of 15% acrylamide:bis 19:1, 8 M Urea and TBE buffer (100 mM Tris, 90 mM boric acid, 1 mM EDTA). Gels were cast at 0.75 mm or 1.5 mm thickness for <200 nmol and >200 nmol RNA respectively. Gels were run in 1x TBE using the C.B.S Scientific Large Format Vertical gel system (C.B.S Scientific), with 35 W for one 0.75 mm gel, or 60 W and 80 W for one or two 1.5 mm gels respectively. Dye mix used was the same as for analytical dPAGE. Gels were imaged with UV shadowing and bands excised. Gel pieces were manually crushed and extracted in 1x TEN buffer (10 mM Tris-HCl pH 8.0, 1 mM EDTA pH 8.0, 300 mM NaCl). The extract was filtered with 0.2 µm CA filters (Whatman 6901-2502) and concentrated to ~ 500 µM in 3 kDa cutoff Amicon concentrators (Millipore), EtOH precipitated by 15 minute incubation in liquid nitrogen, followed by pelleting at 19000 x g for 30 minutes at 4 °C. Pellets were further desalted to desired buffer in 3 kDa Amicon concentrators (Millipore) to ~ 1:10000 dilution factor.

Ion exchange HPLC

Prior to in-cell NMR experiments, dPAGE purified circH44-top was HPLC purified on a preparative DNAPac PA200 9 × 250 column (Thermo Scientific), at 8 mL/min flow rate at a pressure of ~260 bar. With equilibration buffer A (20 mM NaOAc, 20 mM $NaClO_4$, 10% ACN, pH 6.5)) and the elution buffer B (20 mM NaOAc, 600 mM $NaClO_4$, 10% ACN, pH 6.5).

Injections were performed at 2 mL/min column flow rate of <4 mL injection volume with ~ 200 nmol RNA were followed by gradients at 8 mL/min (where %B = 100% - %A): 100% A at 0-2.55 mins, 100-83% A at 2.55-2.72 mins, 83-64% A at 2.72-4.76 mins, 64-0% A at 4.76-5.44 mins, 0% A at 5.44-7.14 mins, 0-100% A at 7.14-15 mins. Fractions were collected as measured by 260 nm absorbance, and concentrated/buffer exchanged in 3 kDa Amicon concentrators (Millipore) to MilliQ H_2O to dilution factor ~ 1:10000.

Confocal microscopy

Cell sample of 100,000 cells were plated onto 24-well µ-Plate Glass Bottom (Ibidi 82427) in 1 mL of L15-, and two drops of Hoechst 33342 (NucBlue Live ReadyProbes Reagent, Invitrogen R37605) added. The plate was incubated for 25 minutes protected from light before analysis. The LSM 700 AxioObserver (Zeiss) confocal laser scanning microscope was used with equipped with Plan-Apochromat 20x/0.8 M27 objective for imaging (BioVis, Scilifelab). Hoechst 33342 and fluorescein were excited with 405 and 488 nm lasers, and their emission was detected with 420–480 and LP490 beamsplitter, respectively. Multitrack mode was used to eliminate the crosstalk between channels. The pinhole parameter was set to 1 Airy unit. Quantification of cellular and nuclear ratios, as well as ratios of RNA signal inside and outside the nucleus was performed in Fiji, as previously, for n=14 cells (ref).

Flow cytometry

500,000 cells were collected: (1) in the first DPBS resuspension follow cell harvest, (2) after electroporation (second wash), and (3) after in-cell NMR. Cells were diluted to 100,000 in 100 µL L15- and 5 µL of 7-AAD (Tonbo Biosciences, 13-6993-T200) was added. Additional samples for compensation calculation were prepared including untransfected cells without 7-AAD, and transfected cells without 7-AAD. The samples for analysis with 7-AAD were incubated at RT for 20 min, protected from light. Flow cytometry was performed on Cytoflex flow cytometer (Beckman Coulter), with B690/50 and B525/40 excitation and band filters for 7-AAD and FAM respectively (BioVis, Scilifelab). Gates were set to exclude cell debris. Data was compensated for spectral overlap from controls and analysed with Cytexpert.

Supernatant control comparison

Following in-cell NMR of biological replicate 2 the supernatant above the cell pellet equal to the active volume of the NMR coil was removed. This makes the first sample for comparison, the 'cell pellet supernatant'. The interstitial medium around the cell pellet is acquired by

inverting the Shigemi to resuspend the cells in the L15 above the cell pellet equal to the volume of the active coil, followed by re-pelleting of cells. The exchanged supernatant was then removed and makes the interstitial medium control.

1D ^{1}H NMR and 2D ^{1}H

1D excitation sculpting experiments with gradients were performed for *in vitro* analysis of RNAs (2). All ^{1}H 1D SOFAST in-cell spectra were acquired as described previously (3). Selective excitation of the imino protons was performed using a PC9 (Pc9_4_120.1000) and an angle of 120°. The selective refocusing was 3.23 ms long (Reburp.1000, 3 ppm bandwidth) (4). The carrier frequency was set to 12 ppm. Gradients for coherence selection in the selective echo as well as a purging gradient before excitation had durations of 1 ms and strengths of 11% and 7%, respectively. The number of scans were 4096 for all experiments, with a D1 of 100 ms and acquisition time of 77.5 ms. Total experimental time for each spectra was 15 minutes and 21 seconds for in-cell spectra. For the shorter recovery delay comparison, a spectra with a D1 of 10 ms was acquired, with acquisition time of 77.5 ms, for 6861 scans and 15 minutes 18 seconds.

^{1}H, ^{1}H Imino NOESY spectra were acquired with mixing times of 175 ms for circmiR34a:Sirt1, 220 ms for circH44-top, 180 ms for circ/GU, and 180 ms for circGUG. For circGUG with 2048 and 128 FID points in the F2 and F1 dimension, 1024 and 256 for cmiR34a:Sirt1, 2048 and 512 for circGU, 2048 and 128 for GU, and 4096 and 640 for circH44-top, respectively.

$R_{1\rho}$ fit equations

Two-state reduced:

$$\Delta\omega_{ES} = \Omega_{ES} - \Omega_{GS} \quad (1)$$

$$\omega_{eff}^2 = \Omega^2 + \omega_{SL}^2 \quad (2)$$

$$\Omega = \Omega_{obs} - \omega_{rf} \quad (3)$$

$$\theta = \frac{\pi}{2} - \arctan\left(\frac{\Omega}{\omega_{SL}}\right) \quad (4)$$

$$R_{eff} = R_2 + \frac{p_A p_B \Delta\Omega^2 k_{ex}}{k_{ex}^2 + w_{eff}^2} \quad (5)$$

Two-state normal:

$$R_{1rho} = R_1 \cos^2 \theta + R_2 \sin^2 \theta + \frac{\sin^2 \theta p_{GS} p_{ES1} \Delta\omega^2 k_{ex1}}{\frac{\omega_{GS}^2 \omega_{ES1}^2}{\omega_{eff}^2} + k_{ex1} - \sin^2 \theta p_{GS} p_{ES1} \Delta\omega_{ES1}^2 (1 + \frac{2k_{ex1}^2 (p_{GS} \omega_{GS}^2 + p_{ES1} \omega_{ES1}^2)}{\omega_{GS}^2 \omega_{ES1}^2 + \omega_{eff}^2 k_{ex1}^2}} \quad (6)$$

Supporting Results

In-cell NMR controls

Flow cytometry and confocal microscopy controls were performed to assess the transfection efficiency and cell viability (93% transfected and viable cells for the cuvette containing fluorescently labelled used in biological replica 1, with 82% viability for all cuvettes combined in biological replica 1, and 87% viability for biological replicate 2 (Supp. Fig. 4-8). The subcellular localisation of the introduced RNA (Supp. Fig. 5) was determined to be in a ratio of 27:73 % (± 23%) nucleus:cytoplasm (Supp. Fig. 4, 5) (3). After two hours of measurement time the medium was replenished as part of the supernatant control. Following 5 and 7.5 hours of in-cell NMR, for biological replicates 1 and 2, the viabilities remained high at 89% and 76%. The supernatant control was performed to assess the proportion of the signal arising from the intracellular RNA versus the surrounding medium. Different tested procedures provided 14.5% and 17% signal arising from the interstitial medium (3,5,6), at 2.6 and 7 hours respectively, versus the classical supernatant control showed 0% and 7%, at 2.6 and 7 hours respectively (Supp. Fig. 9). ^{1}H-SOFAST spectra were acquired in-cell and revealed that the secondary structure of circH44-top remained the same as *in vitro*, with broader linewidths as expected for in-cell experiments (Fig. 4. A, Supp. Fig. 10).

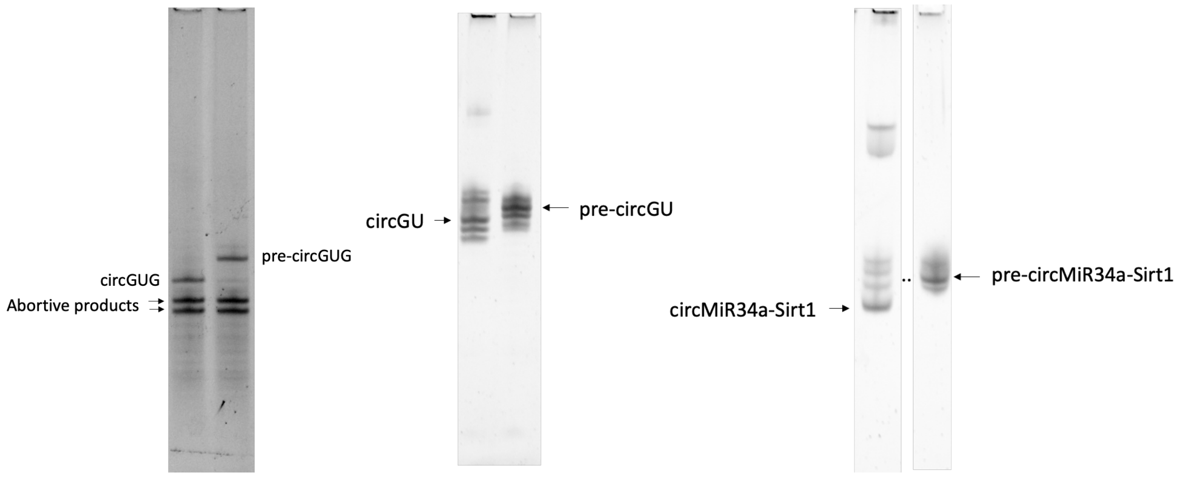

Supplementary Figure 1: Ligation for circmiR34a-Sirt1 and circGU-Tautomer as determined by 20% dPAGE. Band shift is indicative of circularisation (7,8), and is confirmed by NMR, Figure 2.

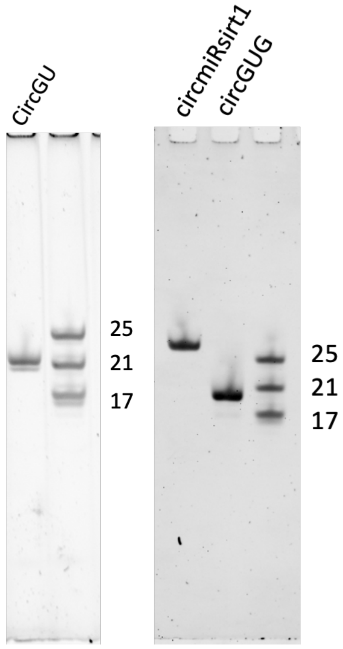

Supplementary Figure 2: Purity determined by 20% dPAGE of circGU, circmiRsirt1, and circGUG used for NMR analysis. circH44-top is represented in Figure 1.

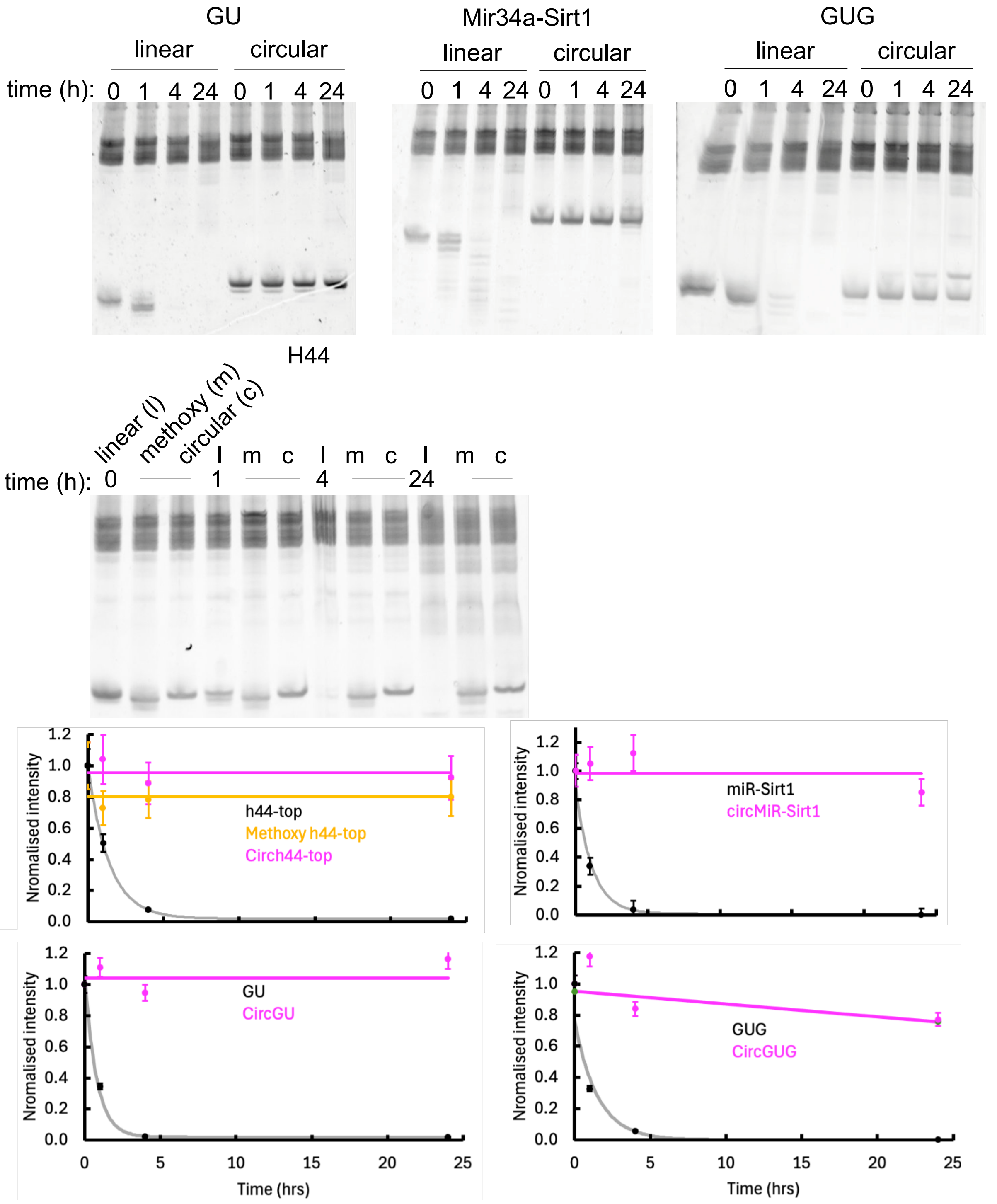

Supplementary Figure 3: Top: 20% dPAGE gels of HEK cell lysate with 37 µM incubated linear and circular RNA. Bottom: Fits for exponential sample decay, $I = I_0 * \exp(R * t)$ in HEK293T lysate as determined from dPAGE. Lanes in Figure 1C quantified using ImageLab. We observe a significant increase in half-life for all circular RNAs beyond the 24 hrs.

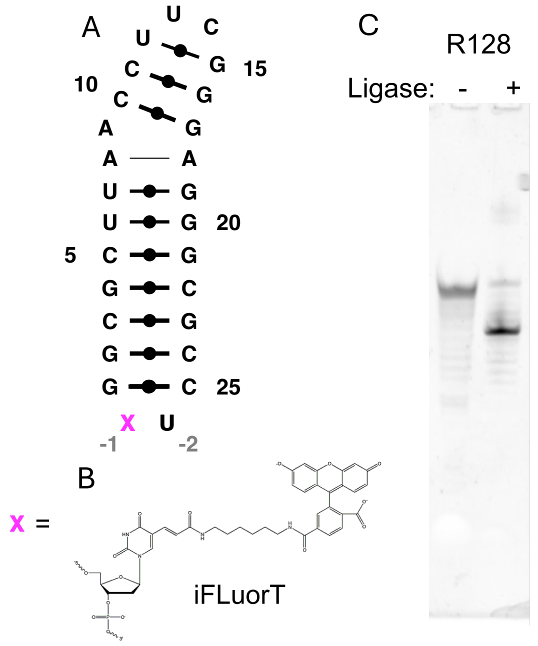

Supplementary Figure 4: A: Secondary structure and circularisation strategy of the fluorescently labelled circH44-top using iFluorT (B) purchased from IDT. The location of the fluorescent modification site was chosen to minimally disrupt the terminal CUUG loop (11). C: Band shift observed on 15% dPAGE indicative of successful circularisation. Fluorescent circH44-top is required for control experiments for in-cell NMR (12)

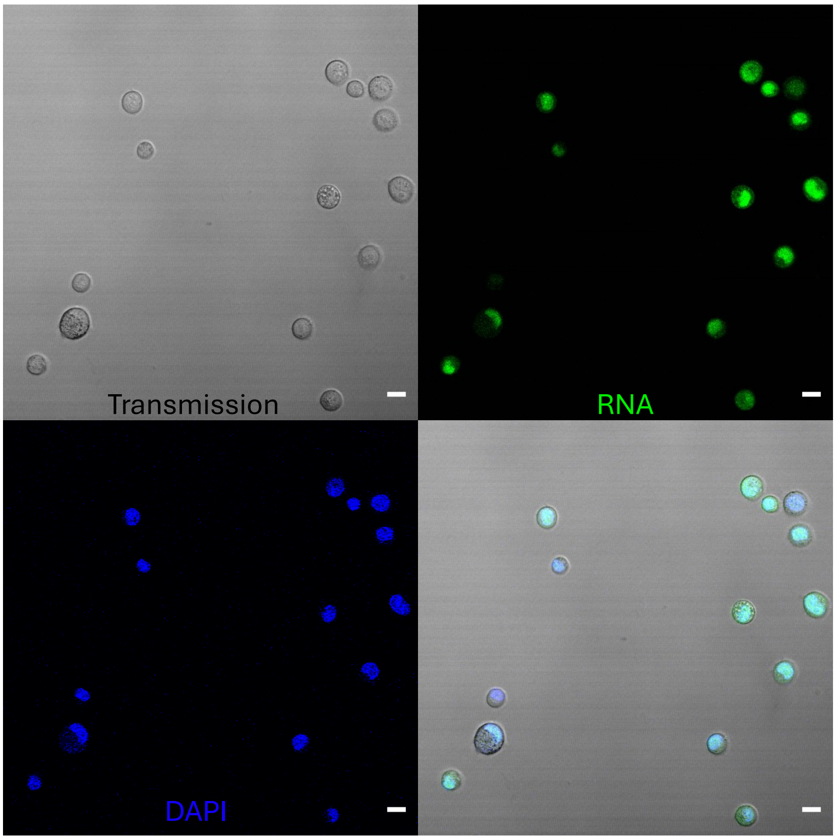

Supplementary Figure 5: Confocal microscopy of HeLa (CCL-2) transfected with circh44-top. HeLa cells were electroporated in a cuvette with buffer (140 mM NaP pH 7.0, 10 mM $MgCl_2$, 5 mM KCl) containing 400 µM circh44-top and 10.25 µM of fluorescein labelled circH44-top. Top left: Transmission image of transfected cells. Top right: fluorescein localization inside the cells. Bottom left: Cell nuclei stained with Hoechst 33342. Bottom right: Overlay of all filters. Images acquired on Zeiss LSM 700 and processed in ImageJ. The concentration of RNA inside the nucleus is higher than in the cytoplasm, but due to the larger cytoplasmic volume the quantity is 27:73 ± 23 % between the nucleus:cytoplasm.

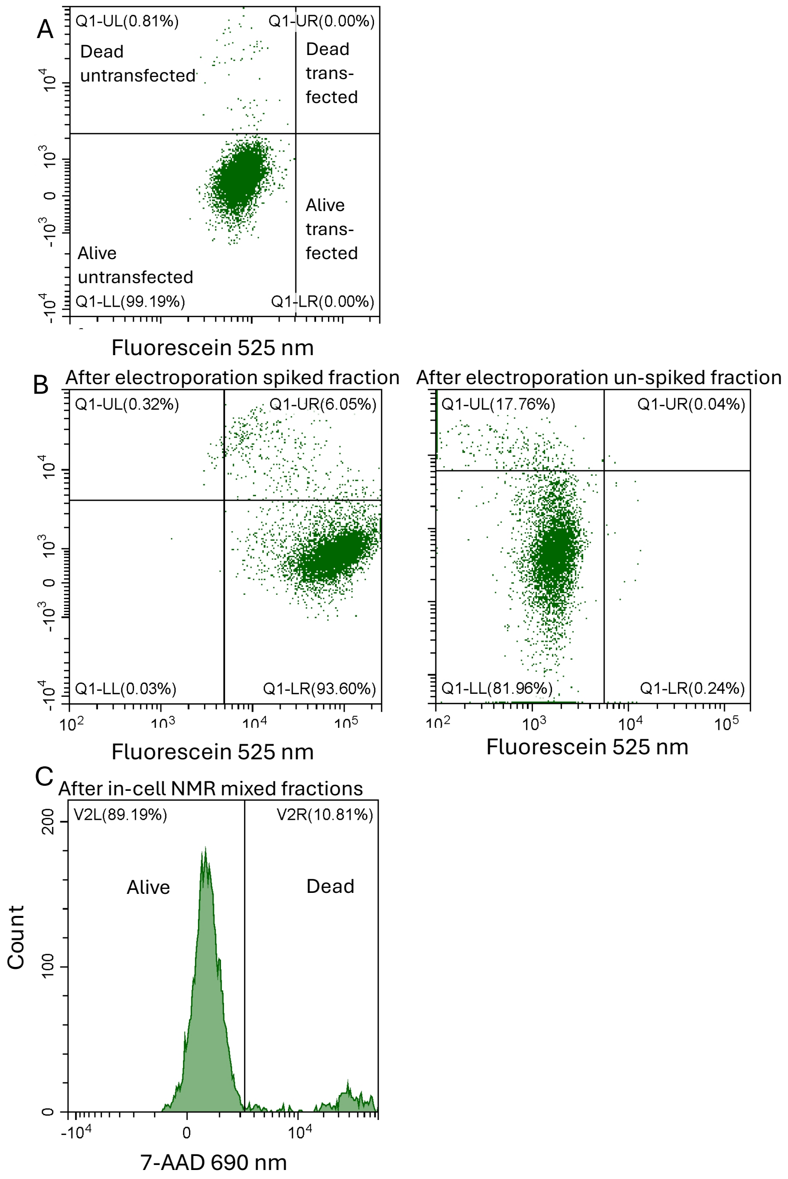

Supplementary Figure 6: Flow cytometry control for and transfection efficiency for in-cell NMR biological replicate 1. A: before electroporation, with quadrants labelled B: After electroporation for the fluorescein containing cuvette (left) and remaining cuvettes without fluorescein labelled RNA (right). C: Viability after 4 hours of in-cell NMR of mixed fractions Cell viability assessed with 7-AAD. Data acquired with 10,000 events on Cytoflex LX2. Gates set to exclude debris. Data processed in Cytexpert. High viability and transfection efficiency is observed following electroporation, and following 4 hours of in-cell experiments.

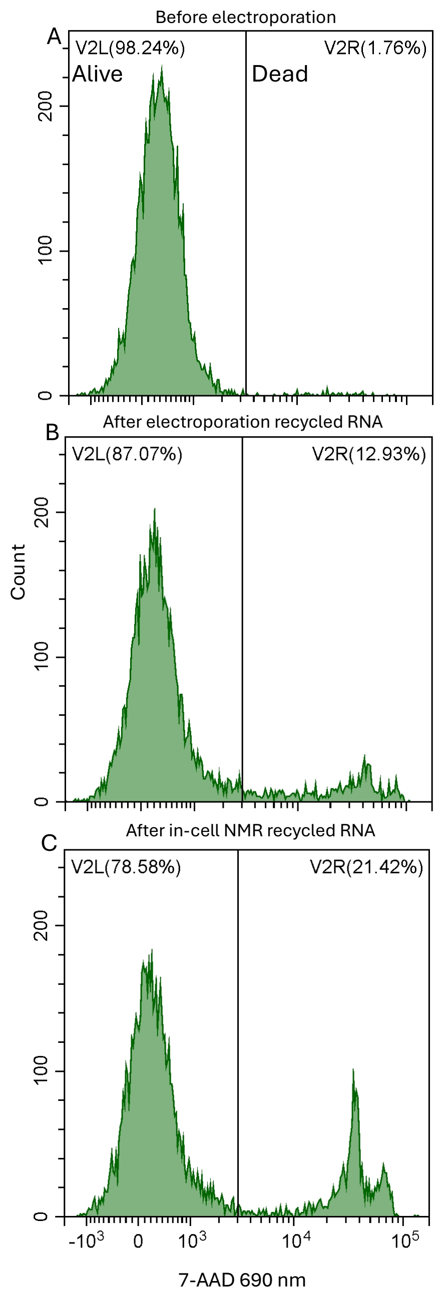

Supplementary Figure 7: Flow cytometry control for viability for in-cell biological replicate 2. Cell viability assessed with 7-AAD. Data acquired with 10,000 events on Cytoflex LX2. Gates set to exclude debris. Data processed in Cytexpert. A: Before electroporation, with segment indicators B: After electroporation and C: After 7 hours in-cell NMR. High viabilities are observed in line with biological replicate 1, and even after 7.3 hours of in-cell NMR.

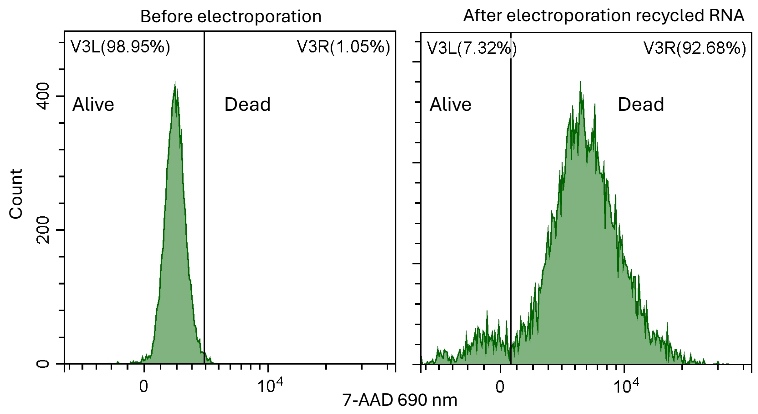

Supplementary Figure 8: Flow cytometry control for biological replicate 2 demonstrating an increase in fluorescence when using recycled iFluorT spiked at 0.4%. However, the shift is not significant enough to quantify accurate transfection efficiency and was therefore excluded from the analysis of transfection efficiency for biological replicate 2 using the recycled RNA.

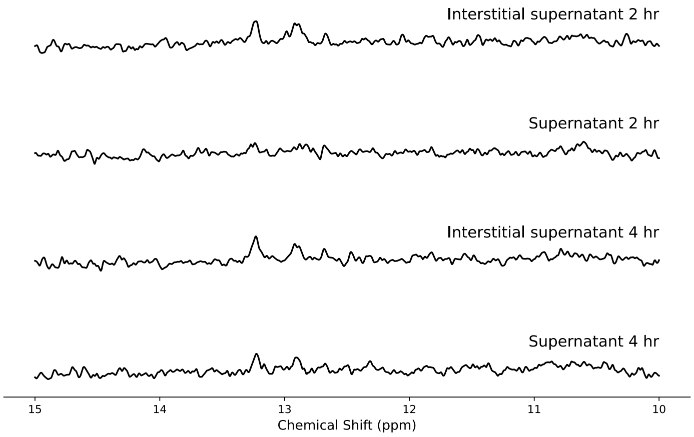

Supplementary Figure 9: Comparison of supernatant preparation methods on biological replicate 2 to assess the suitability of the classical supernatant procedure at recreating the signal arising from the medium surrounding the cell pellet and contributing to the in-cell NMR signal, **Supplementary Materials and Methods**. The interstitial supernatant protocol reveals 14.5% and 17% signal arising from the interstitial medium at 2.6 and 7 hours, versus the classical supernatant control which yields only 0% and 7%. The supernatant procedure under-represents the signal arising from the medium surrounding the cells, and the interstitial protocol should be used.

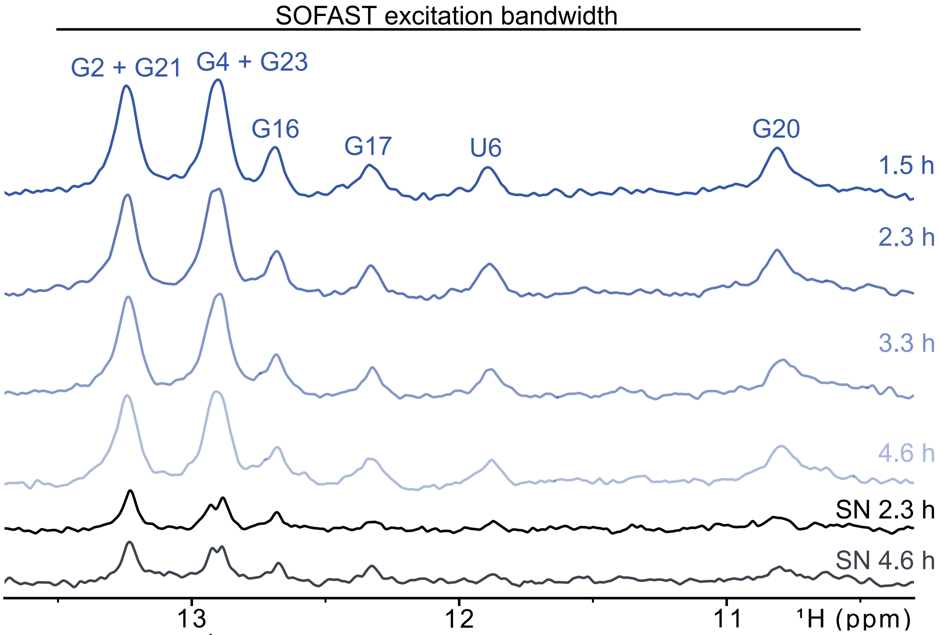

Supplementary Figure 10: 1D-^{1}H SOFAST time course in-cell NMR from biological replicate 1 on circH44-top at 25 °C. 4096 scans per spectra with total acquisition time of 15 minutes and 21 seconds per spectra. Supernatants are interstitial medium. As in Figure 4, circularisation prevents substantial signal decay over the course of 4.6 hours.

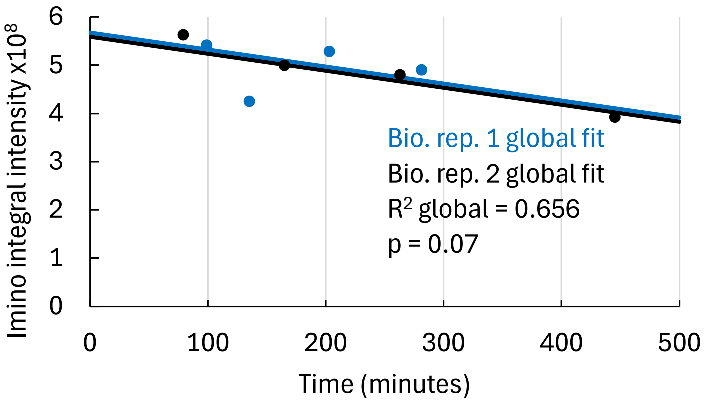

Supplementary Figure 11: Global linear fit with shared decay rate parameter for integrated imino signals from 10-14 ppm in biological replica 1 and 2. Data shown in Figure 4A and Supplementary Figure 15. The signal intensity of circH44-top reduces during the experimental time at -3.7 ± 1.7%/hour.

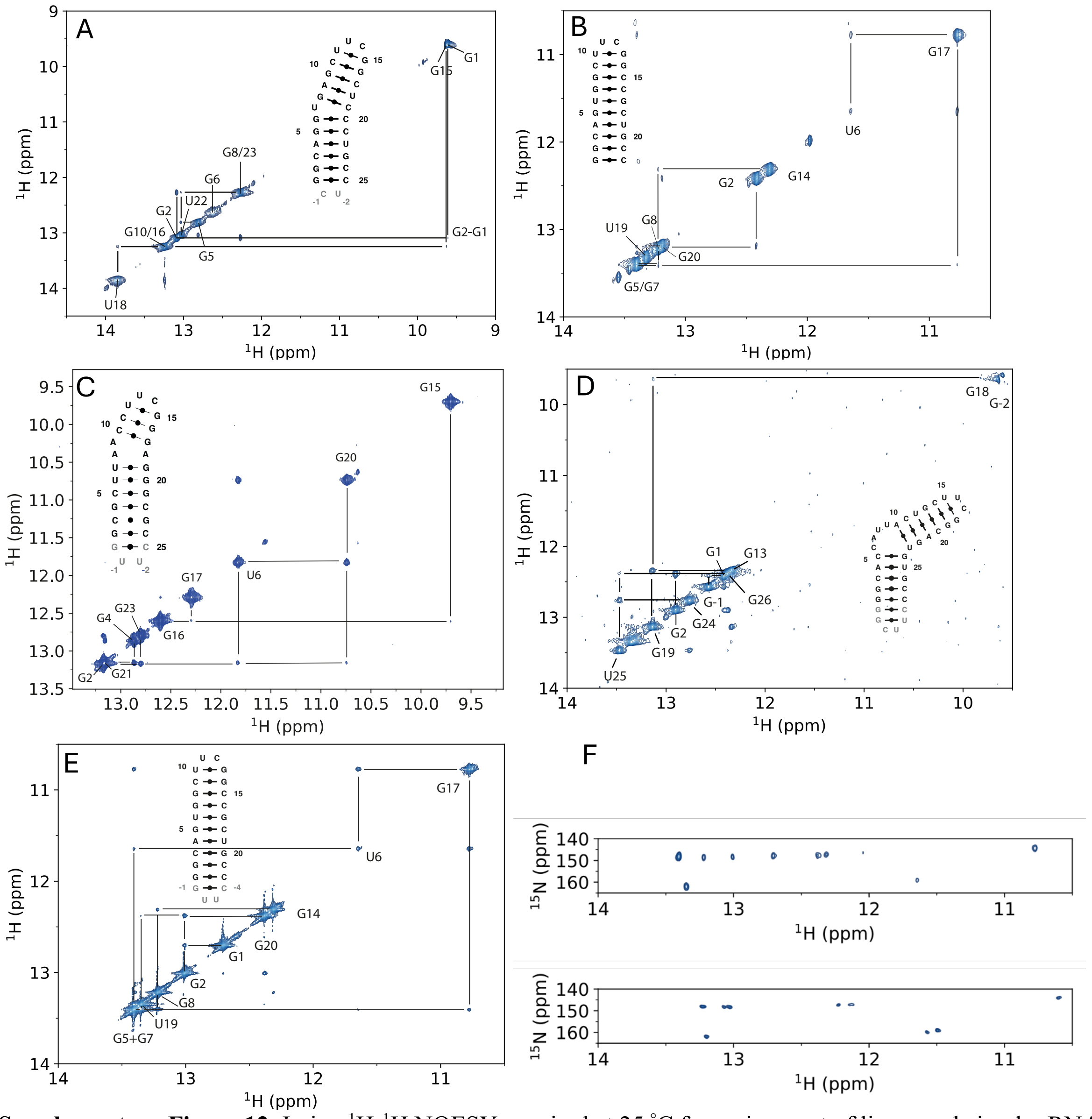

Supplementary Figure 12: Imino ^{1}H-^{1}H NOESY acquired at 25 °C for assignment of linear and circular RNAs. Linear miR34a:Sirt1 is assigned as in (9), and H44-top is assigned as in (10). A: circGUG, B: GU, C: circH44-top, D: circMir34a-Sirt1, E: circGU. F: Bottom is ^{15}N-^{1}H HMQC of linear GU at 5 °C, top is ^{15}N-^{1}H of circular GU at 25 °C. Assignments reveal that linear and circular RNAs contain the same secondary structures.

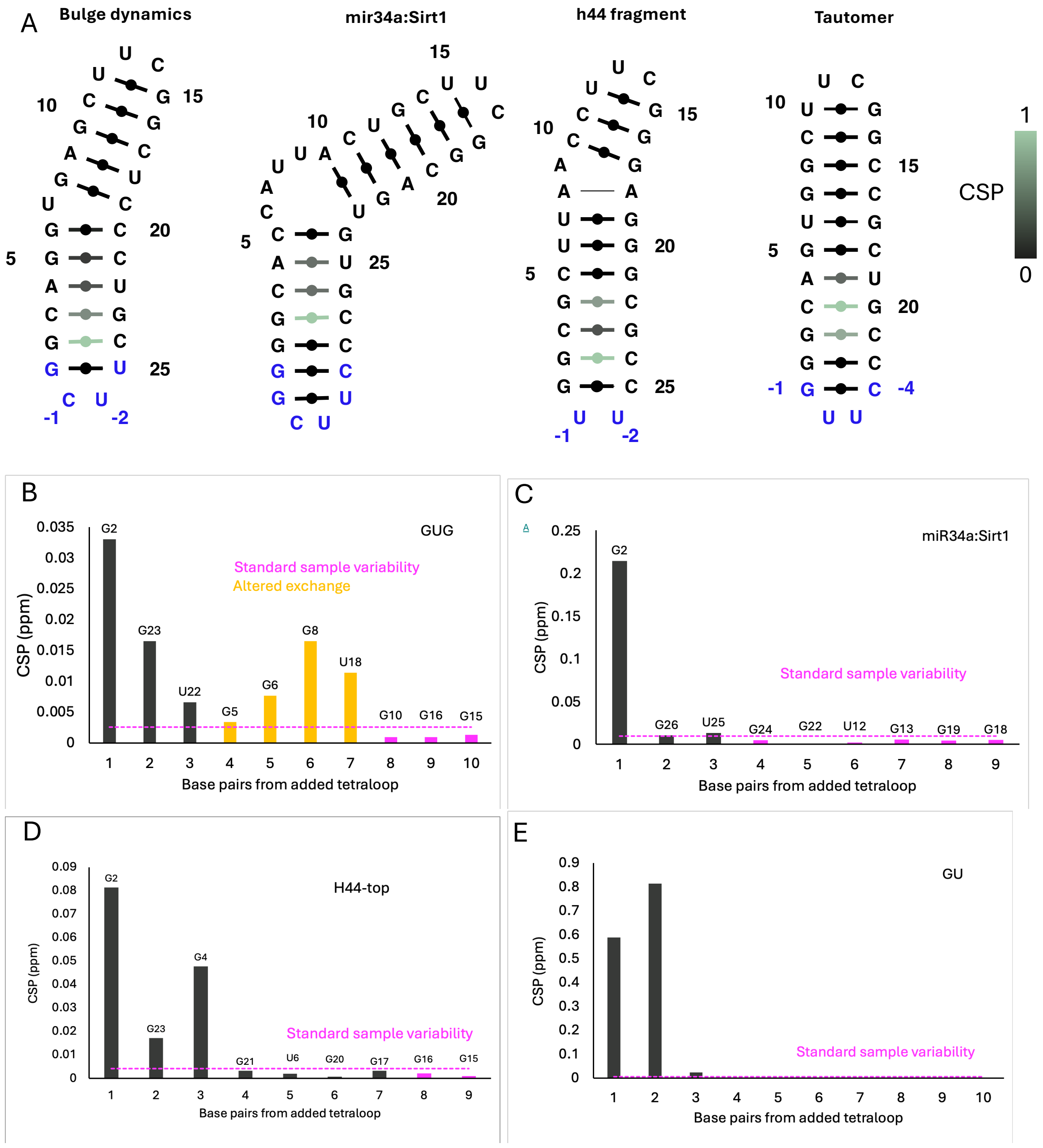

Supplementary Figure 13: A: Chemical shift perturbations (CSP) of imino protons of the four RNA sequences upon addition of the tetraloop. Perturbations are normalised for each construct between 0 and 1, with 1 representing the maximum shift for the construct. The normalised values are plotted on a linear scale, except for miR34a:Sirt1 which is shown logarithmically, due to the large change in G2H1 chemical shift. B-E: Absolute chemical shift perturbation plots with standard sample variability line (pink) that has been determined from 95% confidence interval of standard deviation of original UUCGH1 shifts between linear and circular. Yellow indicates perturbations as a result of altered exchange, and not immediate structural impact. CSP indicates that perturbations are detected up to 3 base pairs away from the added tetraloop, with deviations only for GUG which is shown to be a result of altered dynamics (Supp. Fig. 15).

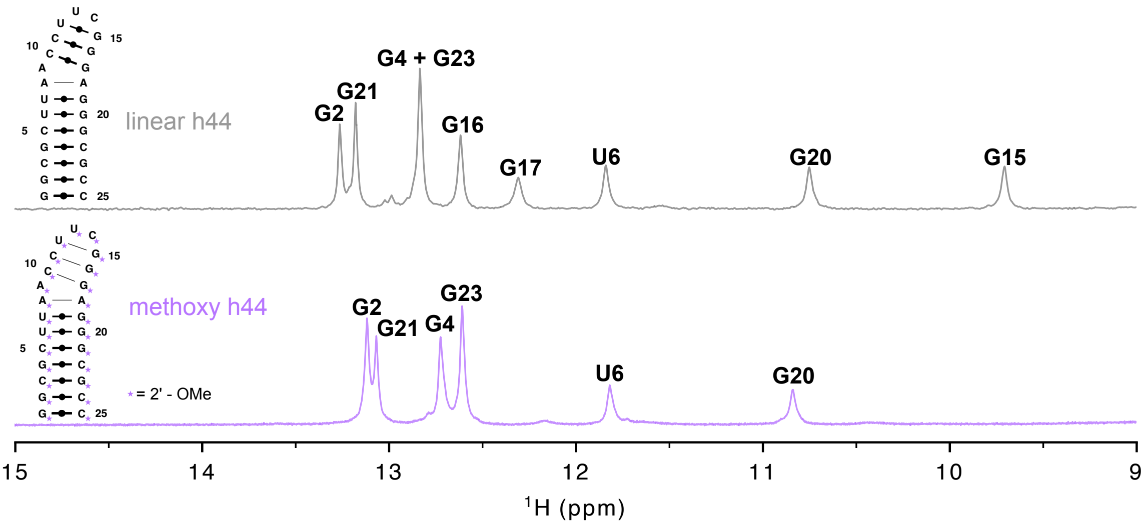

Supplementary Figure 14: 1D-^{1}H imino spectra comparison with secondary structure overlay for unmodified (top) and 2'-O-methylated (indicated by stars) H44-top (bottom) shows that the methoxy modification significantly alters base pair stability in the apical loop. G15 & G16 are not observed, G17 severely broadened indicative of an overall melting of the upper stem loop.

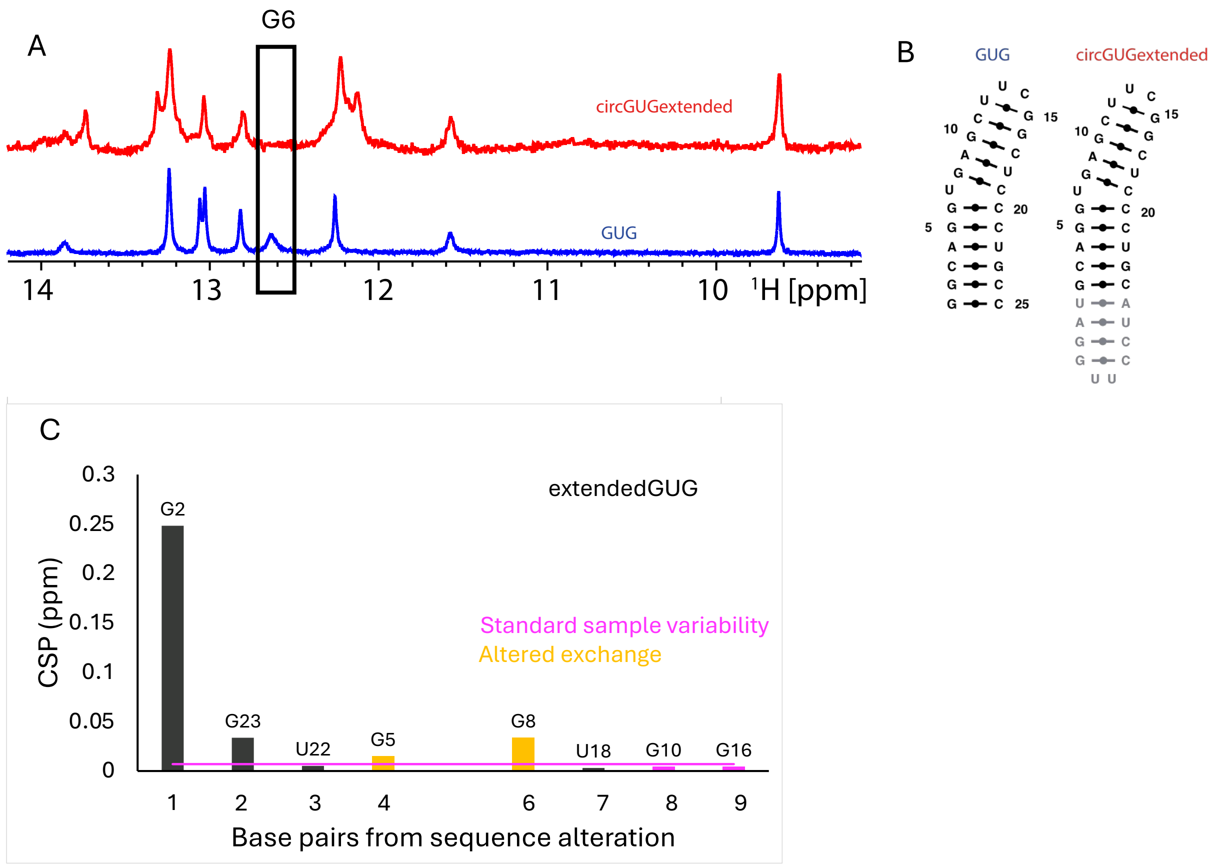

Supplementary Figure 15: A: 1D NMR spectra of imino proton region indicating that the extended circGUG (top, red) still significantly alters dynamics compared to linearGUG (blue bottom), with secondary structures provided in (B). The box highlights the reporter imino proton for dynamics of the neighbouring base pair G6H1. B: Secondary structures of GUG and circGUGextended C: CSP of extended-circGUG G6 is not detectable. Peak assignments are based on chemical shift comparison with linear GUG.

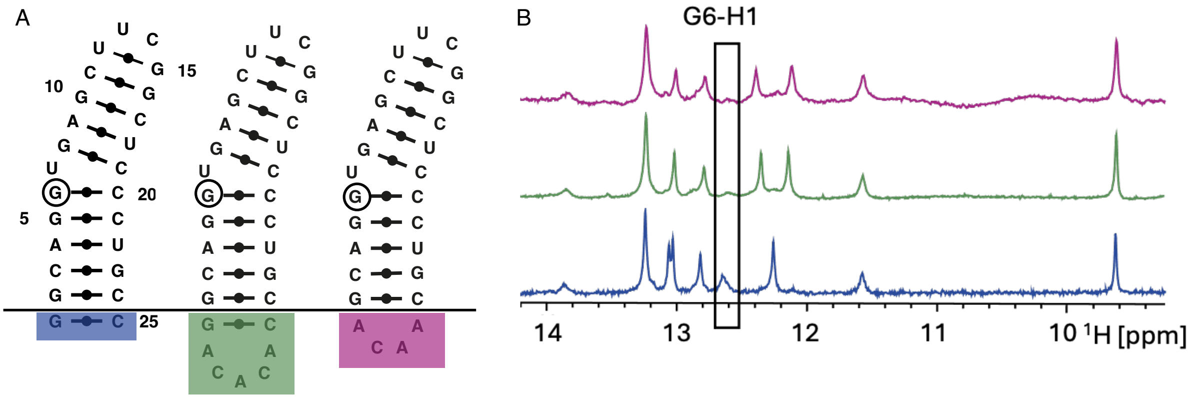

Supplementary Figure 16: (A) Additional loop constructs tested (blue WT) to assess the impact of the less stable tetraloop (AACA violet) and disrupted tetraloop (ACACA green) on dynamics of GUG. (B) ^{1}H spectra overlay of the imino region for the GUG variants with AACA (top, violet) and ACACA (middle, green) closing loops on top of the linear equivalent GUG (bottom, blue). The G6-H1 resonance acts as a reporter for the dynamic bulged uridine (Fig. 3C: bottom of the UUCG-circularised construct using $R_{1\rho}$ relaxation dispersion and Supp. Fig. 15: CUUG-circularised the broadened G6-H1). Broadening of G6-H1 indicates an altered dynamic process following circularisation.

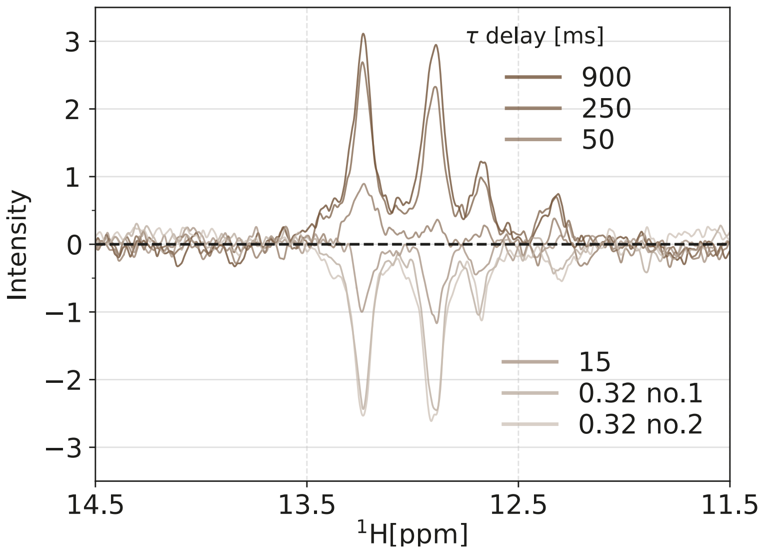

Supplementary Figure 17: Selective inversion recovery for biological replicate 2 to quantify the imino proton selective longitudinal relaxation rate. Figure 4 contains biological replicate 1. Each spectrum acquired with 512 scans for a total experimental time of approx. 19 minutes per spectrum. Data is fit in Supp. Fig. 18 and Fig. 4.

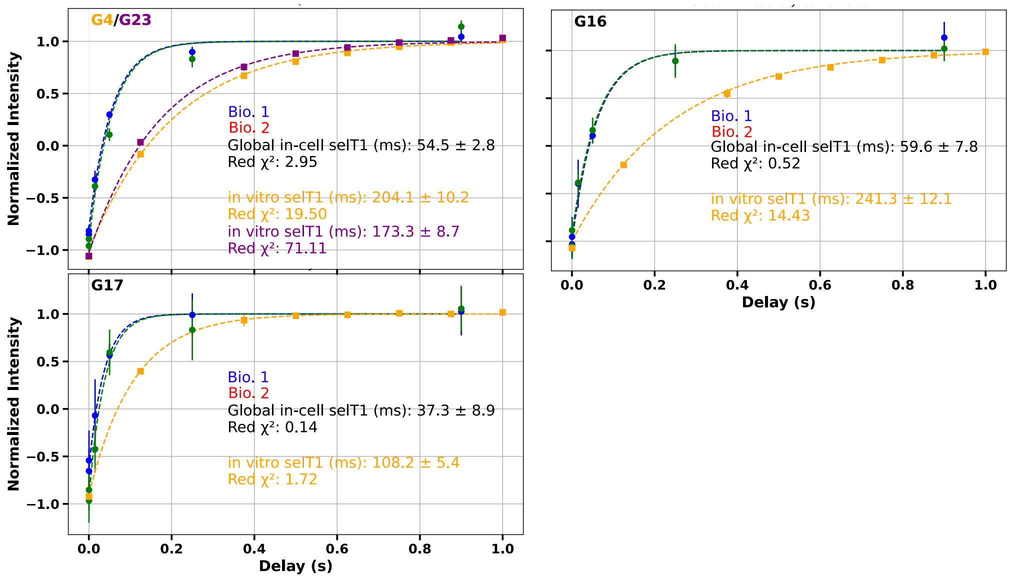

Supplementary Figure 18: Inversion recovery fits for in-cell and in vitro to quantify selT1 in-cell, fitted to I(t) = I(0) (1 – a*exp(-t/selT$_1$)) In-cell fits are globally fitted sharing selT1. This reveals a large reduction in recovery time in-cell compared to in vitro.

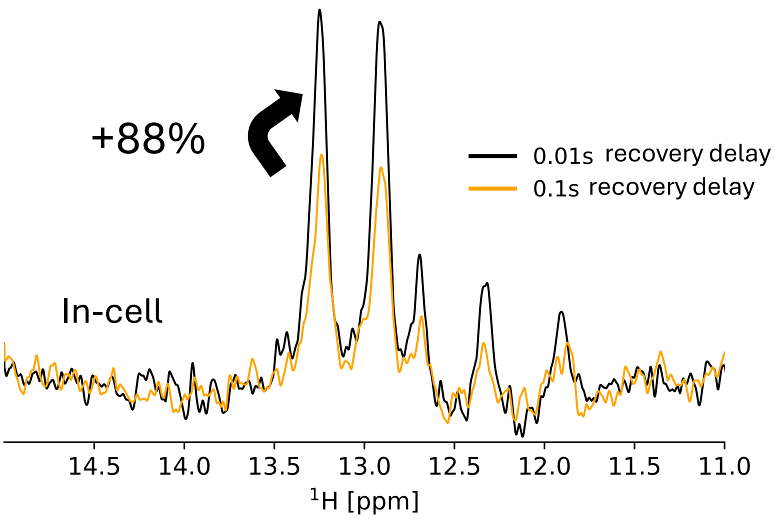

Supplementary Figure 19: ^{1}H-SOFAST in-cell NMR spectra of circH44-top from biological replicate 2 to compare unoptimized and optimized recovery delays based on fitted selT1. For 0.01 s recovery delay the total experiment time was 15 minutes and 18 seconds with 6861 scans. For 0.1 s recovery delay the total experiment time was 15 minutes and 21 seconds with 4096 scans. Overlayed signal increase is determined by integral of the imino region. Significant signal enhancement in made possible by the cellular environment.

Supplementary Table 1: Spin lock strengths and offsets for R_{1r} analysis of all RNAs

**Residue (spin)**	**On-resonance spinlock power (w_1)/ Off-resonance spinlock power (w_1) + (offset (W))**
Circ/MiR34a-Sirt1 G24H1	100, 200, 300, 400, 500, 600,700, 800, 1000, 1200, 1250, 1300, 1500, 1600, 1800, 2000, 2500, 3000, 3500, 4500, 5000, 6000, 8000, 10000, 11000, 14000, 15000
Circ/H44-top G15H1	100, 200, 300, 400, 500, 600, 750, 800, 1000, 1250, 1500, 1600, 1800, 2000, 2200, 2500, 2700, 3000, 3500, 4000, 4500, 5000, 6000, 7000, 8000, 9000, 10000, 11000, 12500, 14000, 15000<br>Off-res:<br>200 Hz (-800, -700, -600, -500, -400, -340, -260, -190, -130, -70, -10, 0, 10, 70, 130, 190, 260, 340, 400, 500, 600, 700, 800)<br>400 Hz (-1600, -1500, -1400, -1300, -1200, -1100, -1000, -900, -800, -700, -600, -500, -400, -300, -200, -100, 0, 100, 200, 300, 400, 500, 600, 700, 800, 900, 1000, 1100, 1200, 1300, 1400, 1500, 1600, 1700)<br>500 Hz (-2000, -1900, -1800, -1700, -1600, -1500, -1400, -1300, -1200, -1000, -800, -600, -400, -200, -125, -50, 0, 50, 125, 200, 400, 600, 800, 1000, 1200, 1300, 1400, 1500, 1600, 1700, 1800, 1900, 2000)<br>800 Hz (-4000, -3500, -3000, -2500, -2000, -1500, -1000, -750, -500, -250, -75, 0, 75, 250, 500, 750, 1000, 1500, 2000, 2500, 3000, 3500, 4000)
Circ/GU U6N1	75, 100, 125, 150, 175, 200, 250, 300, 350, 400, 450, 500, 600, 700, 800, 900, 1000, 1250, 1500, 1750, 2000, 2250<br>Off-res:<br>600 Hz (-2400, -2160, -2040, -1680, -1440, -1200, -960, -720, -600, -480, -360, -240, -120, 0, 120, 240, 360, 480, 600, 720, 960, 1200, 1440, 1680, 2040, 2160, 2400)<br>1000 Hz (-4000, -3600, -3400, -2800, -2000, -1600, -1200, -1000, -800, -600, -400, -200, 0, 200, 400, 600, 800, 1000, 1200, 1600, 2000, 2800, 3400, 3600, 4000)<br>1500 Hz (-6000, -5400, -5100, -4200, -3600, -3000, -2400, -1800, -1500, -1200, -900, -600, -300, 0, 300, 600, 900, 1200, 1500, 1800, 2400, 3000, 3600, 4200, 5100, 5400, 6000)
Circ/GUG G6H1	100, 150, 200, 250, 300, 350, 400, 450, 500, 600, 700, 800, 900, 1000, 1250, 1500, 1750, 2000, 2500, 3000, 4000, 5000, 6000, 7000, 8000, 9000, 10000, 12500, 15000<br>Off-res:<br>200 Hz (-800, -720, -640, -560, -480, -400, -320, -240, -200, -160, -120, -80, -40, 0, 40, 80, 120, 160, 200, 240, 320, 400, 480, 560, 640, 720, 800)<br>400 Hz (-1600, -1440, -1280, -1120, -960, -800, -640, -480, -400, -320, -240, -160, -80, 0, 80, 160, 240, 320, 400, 480, 640, 800, 960, 1120, 1280, 1440, 1600)<br>600 Hz (-2400, -2160, -1920, -1680, -1440, -1200, -960, -720, -600, -480, -360, -240, -120, 0, 120, 240, 360, 480, 600, 720, 960, 1200, 1440, 1680, 1920, 2160, 2400)<br>800 Hz (-3200, -2880, -2560, -2240, -1920, -1600, -1280, -960, -800, -640, -480, -320, -160, 0, 160, 320, 480, 640, 800, 960, 1280, 1600, 1920, 2240, 2560, 2880, 3200)

Supplementary Table 2: Sequences of oligonucleotides

Name	***Sequence 5' – 3'***
GUG	p-GGCAGGUGAGCUUCGGCUCCCUGCC
Mi34a:Sirt1	p-GGCACCAUUACUGCUUCGGCAGUGUGCC
GU	p-GGCAGUGGCUUCGGCCGCUGCC
H44-top	p-GGCGCUUAACCUUCGGGAGGGCGCC
circGUG	p-UUCGGCUCCCUGCUUCGGCAGGUGAGC
circMiR34a:Sirt1	p-GGCACCAUUACUGCUUCGGCAGUGUGCCCUUCGG
circGU	p-GGCUUCGGCCGCUGCCCUUGGGCAGU
circH44-top	p-UUCGGGAGGGCGCCUUGGCGCUUAACC
circGUGextended	p-UUCGGCUCCCUGCaUCcuugGAuGCAGGUGAGC
circH44-top-FAM	p-UUCGGGAGGGCGCCU(iFluorT)GGCGCUUAACC
circH44-top cleavage guide	mCmGmAmA^GGTTmAmAmGmCmGmC
T7 promoter	TTAATACGACTCACTATA
GU template	mGmGCAGCGGCCGAAGCCACTGCCTATAGTGAGTCGTATTAA
H44-top template	mGmGCGCCCTCCCGAAGGTTAAGCGCCTATAGTGAGTCGTATTAA
circGU template	mCmATGCCCAAGGGCAGCGGCCGAAGCCTATAGTGAGTCGTATTA A
circMiR34a:Sirt1 template	mCmCGAAGGGCACACTGCCGAAGCAGTAATGGTGCCTATAGTGAG TCGTATTAA
circH44-top template	TAATACGACTCACTATAGGGAAATAGGGAAATAGCGCUUAACCTTC GGGAGGGCGCCTTGGCGCTTAACCTTCGGGAGGGCGCCTTGGCGC TTAACCTTCGGGAGGGCGCCTTGGCGCTTAACCTTCGGGAGGGCG CCTTGGCGCTTAACCTTCGGGAGGGCGCCTTGGCGCTTAACCTTCG GGAGGGCGCCTTGGCGCTTAACCTTCGGGAGGGCGCCTTGGCGCT TAACCTTCGGGAGGGCGCCTTGGCGCTTAACCTTCGGGAGGGCGC CTTGGCGCTTAACCTTCGGGAGGGCGCCTTGGCGCTTAACCTTCGG GAGGGCGCCTTGGCGCTTAACCTTCGGGAGGGCGCCTTGGCGCTT AACCTTCGGGAGGGCGCCTTGGCGCTTAACCTTCGGGAGGGCGCC TTGGCGCTTAACCTTCGGGAGGGCGCCTTGGCGCTTAACCTTCGGG AGGGCGCCTTGGCGCTTAACCTTCGGGAGGGCGCCTTGGCGCTTA ACCTTCGGGAGGGCGCCTTGGCGCTTAACCTTCGGGAGGGCGCCT TGGCGCTTAACCTTCGGGAGGGCGCCTTGGCGCTTAACCUUCG

p = 5' phosphate
m = 2'O-methyl RNA
^ = cleavage site

References

1. Karlsson H, Baronti L, Petzold K. A robust and versatile method for production and purification of large-scale RNA samples for structural biology. RNA. 2020;26(8). doi:10.1261/RNA.075697.120
2. Hwang TL, Shaka AJ. Water Suppression That Works. Excitation Sculpting Using Arbitrary Wave-Forms and Pulsed-Field Gradients. J Magn Reson A. 1995;112(2). doi:10.1006/jmra.1995.1047
3. Annecke HTP, Eidelpes R, Feyrer H, Ilgen J, Gürdap CO, Dasgupta R, et al. Optimising in-cell NMR acquisition for nucleic acids. J Biomol NMR. 2024 Dec 1. doi:10.1007/s10858-024-00448-5
4. Geen H, Freeman R. Band-selective radiofrequency pulses. Journal of Magnetic Resonance (1969). 1991;93(1). doi:10.1016/0022-2364(91)90034-Q
5. Burz DS, Breindel L, Shekhtman A. Improved sensitivity and resolution of in-cell NMR spectra. In: Methods in Enzymology. 2019. doi:10.1016/bs.mie.2019.02.029
6. Leeb S, Yang F, Oliveberg M, Danielsson J. Connecting Longitudinal and Transverse Relaxation Rates in Live-Cell NMR. Journal of Physical Chemistry B. 2020;124(47). doi:10.1021/acs.jpcb.0c08274
7. Tabak HF, van der Horst G, Smit J, Winter AJ, Mul Y, Koerkamp GMJ a. Discrimination between RNA circles, interlocked RNA circles and lariats using two-dimensional polyacrylamide gel electrophoresis. Nucleic Acids Res. 1988;16(14). doi:10.1093/nar/16.14.6597
8. Chen H, Cheng K, Liu X, An R, Komiyama M, Liang X. Preferential production of RNA rings by T4 RNA ligase 2 without any splint through rational design of precursor strand. Nucleic Acids Res. 2020;48(9). doi:10.1093/nar/gkaa181
9. Baronti L, Guzzetti I, Ebrahimi P, Friebe Sandoz S, Steiner E, Schlagnitweit J, et al. Base-pair conformational switch modulates miR-34a targeting of Sirt1 mRNA. Nature. 2020;583(7814). doi:10.1038/s41586-020-2336-3
10. Steinmetzger et. al. Structural Dynamics of A-minors Regulate Ribosomal Function. Article Submitted. 2026.
11. Jucker FM, Pardi A. Solution Structure of the CUUG Hairpin Loop: A Novel RNA Tetraloop Motif. Biochemistry. 1995;34(44). doi:10.1021/bi00044a019
12. Viskova P, Krafcik D, Trantirek L, Foldynova-Trantirkova S. In-Cell NMR Spectroscopy of Nucleic Acids in Human Cells. Curr Protoc Nucleic Acid Chem. 2019. doi:10.1002/cpnc.71 PubMed PMID: 30489693.
